# Development of a human mitochondrial oligonucleotide microarray (h-MitoArray) and gene expression analysis of fibroblast cell lines from 13 patients with isolated F_1_F_o _ATP synthase deficiency

**DOI:** 10.1186/1471-2164-9-38

**Published:** 2008-01-25

**Authors:** Alena Čížková, Viktor Stránecký, Robert Ivánek, Hana Hartmannová, Lenka Nosková, Lenka Piherová, Markéta Tesařová, Hana Hansíková, Tomáš Honzík, Jiří Zeman, Petr Divina, Andrea Potocká, Jan Paul, Wolfgang Sperl, Johannes A Mayr, Sara Seneca, Josef Houštĕk, Stanislav Kmoch

**Affiliations:** 1 Center for Applied Genomics, 1st Faculty of Medicine, Charles University, Prague, Czech Republic; 2 Institute of Inherited Metabolic Disorders, 1st Faculty of Medicine, Charles University, Prague, Czech Republic; 3 Department of Pediatrics, 1st Faculty of Medicine, Charles University, Prague, Czech Republic; 4 Institute of Molecular Genetics, Academy of Science of the Czech Republic, Prague, Czech Republic; 5 Department of Bioenergetics, Institute of Physiology, Academy of Science of the Czech Republic, Prague, Czech Republic; 6 Department of Pediatrics, Paracelsus Medical University, Salzburg, Austria; 7 Center of Medical Genetics, Free University Brussels, Brussels, Belgium

## Abstract

**Background:**

To strengthen research and differential diagnostics of mitochondrial disorders, we constructed and validated an oligonucleotide microarray (h-MitoArray) allowing expression analysis of 1632 human genes involved in mitochondrial biology, cell cycle regulation, signal transduction and apoptosis. Using h-MitoArray we analyzed gene expression profiles in 9 control and 13 fibroblast cell lines from patients with F_1_F_o _ATP synthase deficiency consisting of 2 patients with mt9205ΔTA microdeletion and a genetically heterogeneous group of 11 patients with not yet characterized nuclear defects. Analysing gene expression profiles, we attempted to classify patients into expected defect specific subgroups, and subsequently reveal group specific compensatory changes, identify potential phenotype causing pathways and define candidate disease causing genes.

**Results:**

Molecular studies, in combination with unsupervised clustering methods, defined three subgroups of patient cell lines – M group with mtDNA mutation and N1 and N2 groups with nuclear defect. Comparison of expression profiles and functional annotation, gene enrichment and pathway analyses of differentially expressed genes revealed in the M group a transcription profile suggestive of synchronized suppression of mitochondrial biogenesis and G1/S arrest. The N1 group showed elevated expression of complex I and reduced expression of complexes III, V, and V-type ATP synthase subunit genes, reduced expression of genes involved in phosphorylation dependent signaling along MAPK, Jak-STAT, JNK, and p38 MAP kinase pathways, signs of activated apoptosis and oxidative stress resembling phenotype of premature senescent fibroblasts. No specific functionally meaningful changes, except of signs of activated apoptosis, were detected in the N2 group. Evaluation of individual gene expression profiles confirmed already known *ATP6/ATP8 *defect in patients from the M group and indicated several candidate disease causing genes for nuclear defects.

**Conclusion:**

Our analysis showed that deficiency in the ATP synthase protein complex amount is generally accompanied by only minor changes in expression of ATP synthase related genes. It also suggested that the site (mtDNA vs nuclear DNA) and the severity (ATP synthase content) of the underlying defect have diverse effects on cellular gene expression phenotypes, which warrants further investigation of cell cycle regulatory and signal transduction pathways in other OXPHOS disorders and related pharmacological models.

## Background

Mitochondria generate most of the cellular energy in the form of ATP, regulate cellular redox state, cytosolic concentration of Ca^2+^, are a source of endogenous reactive oxygen species, and integrate many of the signals for initiating apoptosis. By means of retrograde signaling mitochondria communicate all these events to the nucleus and thus modulate nuclear gene expression and cell cycle.

In humans, mitochondrial dysfunction leads to a vast array of pathologies, and hundreds of diseases result from various defects of mitochondrial biogenesis and maintenance, respiratory chain complexes, or individual mitochondrial proteins [1].

The most frequent group of mitochondrial diseases results from genetic defects of the oxidative phosphorylation system (OXPHOS) [2]. OXPHOS defects form a highly diverse group of diseases that affect primarily energy demanding tissues, such as the central nervous system, heart, and skeletal muscles. Their prevalence is estimated as at least 1:5000 [3]. About half of the OXPHOS defects result from mtDNA mutations [4]. Diseases resulting from mtDNA mutations usually show maternal mode of inheritance and variable penetrance of the disease phenotype, reflecting levels of mtDNA heteroplasmy and threshold effects in affected tissues. Remaining OXPHOS defects result from mutations in genes encoded in nuclear DNA. The majority of the nuclear encoded diseases are inherited as autosomal recessive traits and produce severe and usually fatal phenotypes in infants [5]. Up to now, mutations in approximately 50 nuclear genes have been identified, but most of nuclear genetic defects remain unknown and can involve any of approximately 1000 mitochondria related genes [6]. These genes play an essential role in the assembly or maintenance of individual OXPHOS complexes, in maintenance of mtDNA integrity, and mitochondrial biogenesis.

Diagnostic process of OXPHOS defects requires a combination of biochemical, enzymatic, immunohistochemical and molecular biology methods. To distinguish between isolated and combined OXPHOS deficiencies, the diagnostic process starts with measurements of selected mitochondrial enzyme activities and activities of individual OXPHOS complexes. The diagnostic procedure continues with analysis of OXPHOS complex protein composition. The origin of the molecular defect (mtDNA vs ncDNA) is often apparent from clinical presentation and family history. If not, it can be determined by using transmitochondrial cybrid cell analysis. Final steps in the diagnosis represent mutation analysis either in mtDNA or in nuclear encoded candidate genes in accordance with observed clinical and biochemical phenotypes. The diagnostic process is experimentally demanding and time-consuming and in majority of cases leads only to biochemical diagnosis. The molecular basis of the disease, especially in nuclear encoded defects, mostly remains unknown.

Identification of nuclear gene defects in OXPHOS deficiencies requires combination of positional cloning, functional complementation, and candidate gene analysis. Application of these "standard" procedures is however greatly hampered by limited number of affected patients, complexity and overlap of observed diseases phenotypes, difficulties in measurement of biochemical phenotypes *in vitro*, and by the existence of many candidate nuclear genes [7].

Another method having potential to contribute to differential diagnosis and research of OXPHOS defects relies on gene expression profiling. This type of analysis has a potential to provide information on putative diseases subtypes [8], suggest candidate disease causing genes [7,9,10], reveal pathogenic mechanism of the disease [11] and define specific gene expression profiles usable in future disease class prediction [12].

One of the possibilities for long term studies selectively targeted to mitochondrial gene expression analysis involves development and application of a focused microarray interrogating set of all known and hypothetical human mitochondrial genes, and several human mitochondria focused microarrays were prepared recently [13-16]. All these microarray platforms were based on PCR amplified probes prepared from selected IMAGE consortium cDNA clones. This approach however poses a number of technical obstacles. High rate of miss-annotation and contamination in the commercially distributed subset of the IMAGE Consortium cDNA clone collection [17] requires resequencing of individual clone inserts, and subsequent PCR preparation of individual probes is laborious and time consuming. Given these difficulties it has become very attractive to use sets of oligonucleotide probes that obviate much of the probe preparation work. Since the yield of long oligonucleotides has improved and cost has fallen recently, the current trend in preparation of low density, tailor-made microarrays favours oligonucleotide microarrays [18].

In this paper, we describe development and validation of a focused oligonucleotide microarray for expression profiling of human mitochondria related genes – "h-MitoArray" and report gene expression analysis of fibroblast cell lines from 9 controls and 13 patients with isolated deficiency of F_1_F_o _ATP synthase caused either by microdeletion of mtDNA encoded ATP6 gene [19,20] or by mutation of unknown nuclear genes [21,22].

## Results

### Microarray design and preparation

For microarray preparation we selected genes coding for known or predicted mitochondrial proteins, genes known to be involved in cell cycle growth and regulation, and genes involved in apoptosis and free radical metabolism.

The final set contained 1632 genes, of which 992 are "mitochondrial" genes, 42 lysosomal genes, 277 genes are associated with apoptosis, and 321 are "oncogenes". For normalization and background correction we included 146 human "housekeeping" genes, 10 *Arabidopsis *genes and 32 blanks. Full list of selected genes with corresponding symbols, accession and LocusLink codes is provided [see Additional file 1]. Functional annotation of selected genes and comparison of the gene content against whole human genome set is provided [see Additional file 2].

### Microarray validation

Hybridization properties and performance of designed oligonucleotide probes and control features placed on h-MitoArray were tested by hybridization of fluorescently labeled panomers and fluorescently labeled cDNA prepared from a pool of total RNA isolated from several cell lines (test RNA). Gene expression signal was detected in > 77% of 1820 elements when fluorescently labeled cDNA pool was used.

Following comparison of various labeling strategies and optimization of hybridization conditions a series of self-to-self experiments was performed using test RNA. Data analysis showed acceptable reproducibility with Pearson correlation coefficient ranging 0.987 – 0.991.

### Gene expression analysis in ATP synthase deficient fibroblasts

Fluorescent cDNA probes labeled with Cy5 were prepared from 13 patient and 9 control cell lines and were hybridized to common reference cDNA probe labeled with Cy3 in two technical replicates for each sample. Following data acquisition, transformation, normalization and replicate averaging, gene expression signals were obtained for 1264 genes. Ratios of Log2 sample gene intensities against Log2 common reference gene intensities (M) were calculated and are provided [see Additional file 3]. Calculated ratios of individual patient Log2 gene intensities against the Log2 of average of controls gene intensities (M) are provided [see Additional file 4].

#### Principal component analysis

**Figure 1:**
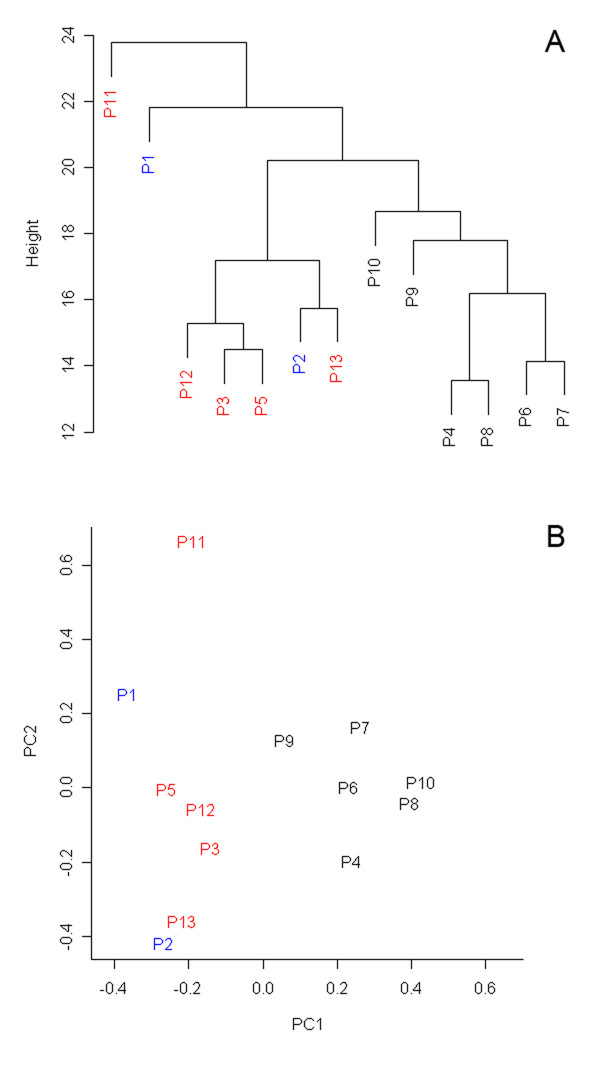
**Results of unsupervised clustering methods**. **A) **Dendrogram resulting from two-dimensional hierarchical clustering of all genes across all patient samples performed using Euclidean distance metrics and average linkage clustering algorithm. **B) **Two-dimensional PCA plot of all expression data showing the separation of samples forming N1 group. Patients from M, N1 and N2 groups are shown in blue, black and red, respectively.

To assess overall data quality and visualize relations between analyzed samples, we removed from the original data set 47 genes showing low expression variability (based on criteria |Mmin; Mmax| ≤ 0.58, less than 1-fold change across all the samples) and subjected resulting data set to principal components analysis. Visual inspection of resulting plots showed no gross differences among the individual samples but suggested that several samples from nuclear defect patients group might be distinct from the others (Figure 1B).

#### Hierarchical clustering

To reveal gene expression changes, survey variation in patient samples, and better interpret the results of principal component analysis (PCA), gene expression signals from individual patient samples were compared to average of gene expression signals from all controls. Hierarchical clustering of all gene ratios across all patient samples was performed using Euclidean distance metrics and average linkage clustering algorithm. Resulting expression map (not shown) and sample dendrogram shown in Figure 1A defined, in agreement with previous PCA, two distinct subgroups of patients with nuclear defect, (N1 and N2 group) which were considered in subsequent gene expression comparisons and functional evaluations.

#### Overall gene expression changes triggered by ATP synthase deficiency

Comparison of gene expression patterns between ATP synthase deficient and control fibroblast cell lines was performed in R statistical environment as described in methods. This analysis revealed 78 genes to be differentially expressed at adjusted P < 0.01 significance level [see Additional file 5]. Detailed inspection of expression map and evaluation of individual gene expression profiles showed, that although defined as significant, majority of the identified genes was not uniformly altered across all the patient samples.

#### Identification of subgroup specific gene expression profiles

**Figure 2:**
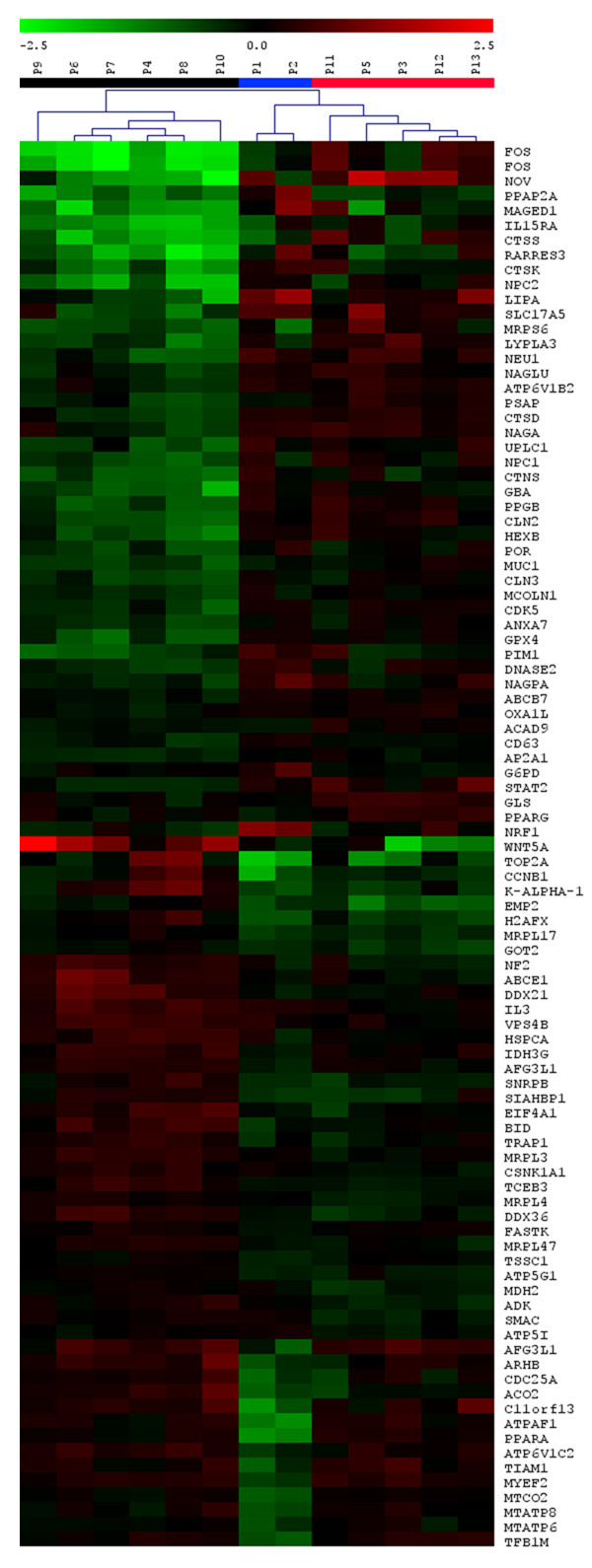
**Differentially expressed genes defined by ANOVA analysis**. Heatmap of genes detected as differentially expressed between defined patient groups using ANOVA analysis and unadjusted P < 0.01 significance level. The results are shown as Log2 ratio of relative gene expression signal in each patient sample to average of this of control samples. Ratio values are represented as the pseudo-color whose scale is shown in corresponding lookup picture.

To identify the subgroup specific gene expression changes, the subgroups of patients defined by a mutation of the *MTATP6 *gene of the mtDNA (M group), PCA and hierarchical clustering (N1 and N2 groups) were compared. ANOVA analysis performed in MeV software revealed 97 genes to be differentially expressed at unadjusted P < 0.01 (Figure 2), [see Additional file 6].

Inspection of resulting data showed that the M group was specifically characterized by reduced expression of mitochondria encoded ATP synthase subunit genes *MTATP6*, *MTATP8*, nuclear encoded ATP synthase assembly factor *ATPAF1*, cytochrome *c *oxidase subunit II gene *MTCO2*, mitochondrial transcription factors *TFAM *and *TFB1M*, peroxisome proliferator-activated receptor alpha (*PPARA*), regulatory genes *H2AFX*, *CCNB1*, *C11orf13 (RASSF7)*, *TPR *and *ACO2*. This was accompanied by induction of *NRF1*.

The N1 group was characterized by reduced expression of genes involved in cell growth, differentiation and transduction pathways (*FOS*, *NOV*, *MAGED1*, *IL15RA*, *RARRES3*, *CTSK*, *UPLC1*, *PIM1*), mitochondrial proteosynthesis (*MRPS5*), lysosomal metabolism and function (cathepsins *S, K *and *D*, *GBA*, *PPGB*, *NPC*, *CLN2*, *FUCA1, HEXB*), protein transport (*AP2A1*), protein phosphorylation (*CDK5*, *PPAP2A*), hydrolase activity (*LIPA, LYPLA3*), reactive oxygen species metabolism (*GPX4*) and membrane transport (*SLC17A5*, *CTNS*). This was accompanied by elevated expression of several cell cycle regulatory genes such *WNT5A*, *IL3*, *CSNK1A1*, *BID*, *EIF4A1*, and *ACO2*.

The N2 group showed reduced expression of *WNT5A*, *EMP2*, *ADK*, *MDH2*, *SMAC *and elevated expression of *PPARG *and *GLS*. Extent and range of detected changes were much less than that observed in M and N1 groups.

Following ANOVA analysis, which revealed only inter-group specific differences, a list of group specific gene expression changes was obtained by comparison between defined patient subgroups and controls in R statistical environment as described in Methods. The analysis revealed 61, 215, and 54 genes to be differentially expressed at adjusted P < 0.01 in the M, N1 and N2 groups, respectively. In addition to the above mentioned genes revealed by ANOVA, we found in the M group elevated expression of *mitofusin *and coordinately reduced expression of genes regulating G1/S phase transitions (*E2F1*, *MYC*, *CDC2*, *GAS1*, *CCNA2*, *CCNB*, *CDK2*, *CDC25A*, *PCNA*), thymidine metabolism (*TK*, *TYMS*) and DNA topology (*H2AFX*, *TOP2*, *LMN2*). In the N1 group, we observed reduced expression of genes regulating cell growth and signaling (*JUNB*, *MAPK3*, *WT1*, *CEBPA*, *CEBPB*) and lysosomal metabolism. We found elevated expression in genes involved in apoptosis (*FAS*, *CYTC*, *SMAC*, *IGFBP3*). In the N2 group, we found signs of started apoptosis (*SMAC*, *CASP8*). Group specific gene lists with expression values and corresponding P-statistics are provided [see Additional file 7, 8, 9].

### Biological consequences of identified gene expression changes

To reveal biological consequences and to identify pathways potentially involved in the pathogenesis of the studied defects, we extracted from original expression data for each of the three defined groups all genes found to be differentially expressed at unadjusted P < 0.05 and showing expression change |M| > 0.2. Resulting expression datasets were uploaded into the DAVID database [23] and gene enrichment analysis was performed against h-MitoArray gene list. Results are provided in Table 1.

**Table 1:** Functional annotation of defined patient subgroups.

**M**			**N1**			**N2**		
category	n	p	category	n	p	category	n	p

**DAVID IDs**								

	258			383			238	

**Biological processes**								

	230			344			203	
DNA replication	13	5E-3	endodome transport	7	1E-3	development	38	9E-3
taxis	9	7E-3	vacuole organization and biogenesis	7	9E-3	reactive oxygen species metabolism	6	1E-2
carbohydrate metabolism	25	9E-3	response to chemical stimuli	23	1E-2	response to oxidatïve stress	5	3E-2
negative regulation of biological processes	26	1E-2	regulation of enzyme activity	20	3E-2	dephosphorylation	6	2E-2
nucleic acid metabolism	69	2E-2	vesicle mediated transport	18	3E-2	intracellular protein transport	18	3E-2

**Molecular function**								

	238			347			211	
DNA binding	39	2E-2	protein dimerization activity	14	2E-2	protein domain specific binding	6	3E-2
protein dimerization activity	10	5E-2	hydrolase activity on glycosyl bonds	12	5E-2	GTPase activity	7	5E-2
nucleic acid binding	54	5E-2						

**Cellular component**								

	285			342			195	
chromosome	11	2E-3	vacuole	44	2E-8	chromosome	8	4E-2
chromatin	7	9E-3	lytic vacuole	39	2E-7			
nucleus	70	6E-3	lysosome	39	1E-7	lytic vacuole	17	4E-2
lytic vacuole	20	2E-3	extracellular region	32	2E-2	lysosome	17	4E-2
lysosome	20	2E-3	endosome	8	4E-2	non-membrane bound organelle	28	4E-2

**KEGG pathway**								

	122			185			125	
N-glycan degradation	5	2E-2	antigen processing	9	2E-3	Toll-like receptor signaling	10	2E-2
hematopoetic cell lineage	8	3E-2	glycosphingolipid metabolism	7	2E-2	glycosylaminoglycan degradation	6	2E-2
			hematopoetic cell lineage	10	4E-2			

**Biocarta pathway**								

	68			92			57	
cyclins and cell cycle regulation	9	2E-2	role of ERB2 in signal transduction	9	5E-3	activation of Src	4	3E-2
			IL 3 signaling pathway	7	1E-2	phospholipid signaling intermediates	5	4E-2
			IL 6 signaling pathway	8	1E-2			
			Erk and PI-3 kinase pathway	7	2E-2			
			signaling pathway from G-protein families	7	3E-2			

"n", number of genes involved in the corresponding annotation category; p, modified Fisher exact p-value of the gene enrichment for each category.

As the enrichment analysis suggested group specific dysregulation of several metabolic and signaling pathways, we further uploaded identical datasets into KEGGArray software (KEGG pathway databases – Kyoto Encyclopedia of Genes and Genomes) and inspected gene expression changes in all the indicated pathways.

**Figure 3:**
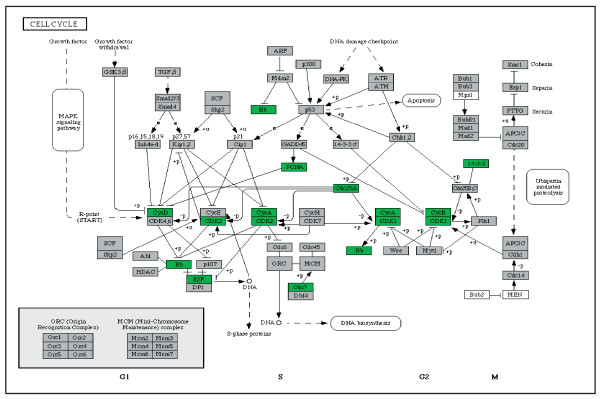
**Gene expression changes detected in selected pathways in M group**. General changes in cell cycle pathway detected in patients with mtDNA mutation (M group) using KEGGArray software.

In the M group, generally reduced expression was observed in cell cycle regulation (Figure 3), Krebs cycle (*OGDH*, *IDH1*, *ACO2*) and gluconeogenesis (*ALDOA*, *LDHA*, *PGAM1*) pathways. With an exception of *MTATP6*, *MTATP8 *and *MTCOX2*, no multiple changes in OXPHOS system, valine, leucine, isoleucine, lysine, β-oxidation and MAP kinase pathway were observed. Reduced expression of *CytC *and *NFκB *and elevated expression of *FAS *were detected in the apoptotic pathway. In contrast to the N1 group, elevated expression of genes involved in N-glycan and heparan sulfate was detected.

**Figure 4:**
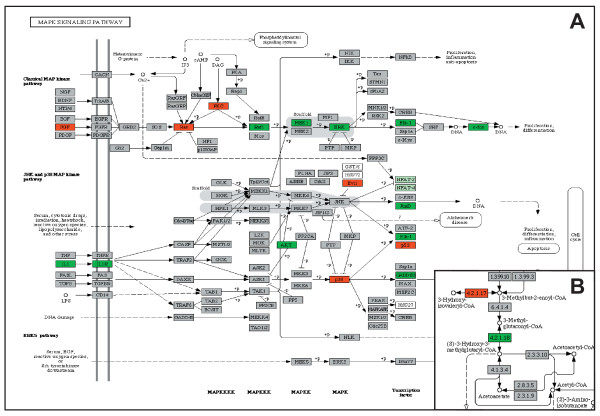
**Gene expression changes detected in selected pathways in N1 group**. **A) **Changes in MAPK, JNK and p38 MAP kinase pathways, **B) **reduced expression of AUH, 3-methylglutaconyl-CoA hydratase gene in leucine degradation pathway, detected in patients with nuclear defect (N1 group) using KEGGArray software.

In the N1 group, the analysis revealed elevated expression of several complex I subunit genes (*ND1*, *ND2*, *ND4*, *ND4L*, *Ndufs1*, *Ndufv2*, *Nufa9*, *Ndufb9 and Ndufa10*) and generally reduced expression of complex IV (*COX4*, *COX5A*, *COX6A*, *COX6B*, *COX6C *and *COX15*) and complex V subunit genes (*ATPAF1*, *ATP5G2*) in OXPHOS system. Generally reduced expression of V-type ATP synthase subunit genes was observed. Elevated transcription activity was found along valine, leucine, isoleucine, lysine and fatty acid β-oxidation pathways. Elevated expression of *FGF*, *FGFR*, *Ras *and *PKC *and reduced expression of *Raf1*, *MEF1*, *ERK*, *Elk1 *and *FOS *were found in classical MAP kinase pathway (Figure 4A). Reduced expression of *IL1*, *IL1R*, *AKT*, *Elk1*, *GADD153 *and *JunD *with elevated expression of *p53, p38 *and *Evi1 *were found in JNK and p38 MAP kinase pathways (Figure 4A). Reduced expression of *STAT, CPB, Pim-1, AKT *and *BclXL *and elevated expression of *IL2/3 *and *IL3R *were found in Jak-STAT signaling pathway. Elevated expression of *Bid *and *CytC *with reduced expression of *Bcl-2/XL *and *CASP9 *were detected in the apoptotic pathway. General decrease in expression of genes involved in N-glycan, glycosylaminoglycan, and ganglioside degradation was found. In conjunction with 3-methylglutaconic aciduria, which is a characteristic biochemical feature of the patients from this group, inspection of leucine degradation pathway showed moderately reduced expression of 3-methylglutaconyl-CoA hydratase gene, *AUH*, (Figure 4B). Although the extent of the *AUH *expression changes neither directly implicates the deficiency of 3-methylglutaconyl-CoA hydratase nor explains 3-methylglutaconic aciduria present in these patients, it is possible that such changes might be much more pronounced and have functional effects during metabolic stress and/or in metabolically active tissues. In the N2 group, reduced expression of *GRB2, RAS *and *ERK *and elevated expression of *FOS, JUND *and *Evi1 *was found in MAP kinase pathway. This was accompanied by elevated expression of genes involved in N-glycan, glycosylaminoglycan and ganglioside degradation. No multiple changes in the apoptotic and valine, leucine, isoleucine, lysine, β-oxidation degradation pathways were found. All mentioned pathways and gene expression changes identified by KEGGArray software are provided [see Additional file 10 and 11].

#### Identification of patient specific gene expression profiles and definition of candidate disease causing genes

**Figure 5:**
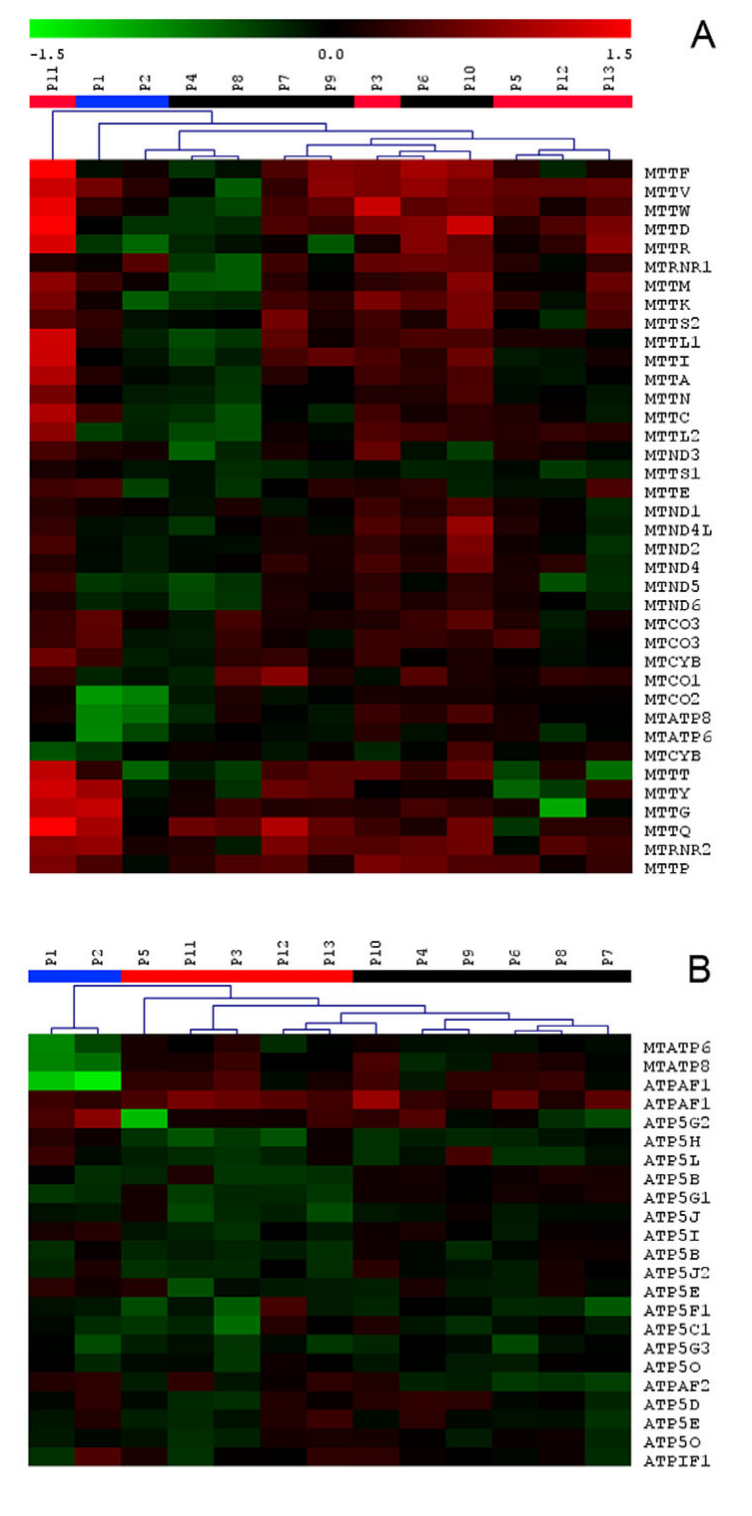
**Two-dimensional hierarchical clustering of patient samples**. **A) **Expression matrix of all 37 mtDNA encoded genes. **B) **Expression matrix of structural and assembly factor genes involved in ATP synthase complex biogenesis. Selected genes were clustered across all patient samples using Euclidean distance metrics and average linkage clustering algorithm.

To get specific information on patient mitochondrial genome expression, we extracted and clustered gene expression data for all 37 mtDNA genes. Resulting mitochondrial genome expression map (Figure 5A) reflects relative mitochondrial DNA amount with generally elevated expression in P11, P3, P10 and P6 and generally reduced expression in P2, P4 and P8. Specific gene expression changes were detected in P1 and P2, where the expression map revealed reduced amount of *MTATP6*, *MTATP8 *and *MTCOXII *transcript reflecting disease causing microdeletion of *MTATP6*, and in P12 with specifically reduced expression of *tRNAGly*.

To obtain the information on patient specific ATP synthase complex expression, we extracted and clustered gene expression data for all of its structural genes and assembly factors. Resulting expression map is provided in Figure 5B. In P1 and P2, it shows reduced expression of mitochondrial subunits *MTATP6, MTATP8 *and also of *ATPAF1*. With the exception of reduced expression of *ATP5G2 *in P5 and maybe also *ATP5C1 *in P3 no additional subgroup and/or patient specific profile were found.

To define potential candidate disease genes, we finally compared gene expression data of individual patients with a group of controls in R statistical environment as described in methods, and searched for genes showing significantly reduced expression and having known function either in ATP synthase biogenesis, mitochondrial protein trafficking or mitochondrial biogenesis. In P1 and P2, we detected reduced expression of ATP synthase structural subunits *MTATP6, MTATP8 *and also of *ATPAF1*. In P3 we detected reduced expression of *ATP5C1 *and *ATP5O*. In P4 and P8 we detected reduced expression of *TOM 7*. Mitochondrial carrier homolog 1 (*C. elegans*) (*MTCH1*) transcript was reduced in P6, P10, P11 and P12.

In P10 we detected reduced expression of mitochondrial elongation factor *EFG1 *and *TOM22*. In P11 we found reduced expression of *TIM23, TIM8 *and *TOM34 *homologs, *ATP5H *and ATP5E. In P12 we found reduced expression of *mitofusin *and *ATP5H*. The lists of all the differentially expressed genes are shown [see Additional file 12, 13, 14, 15, 16, 17, 18, 19, 20, 21, 22, 23, 24].

### Confirmation of the hybridization results

To rule-out platform specific bias, we re-analyzed all RNA samples from the N1 and control groups using the same common reference RNA on Agilent 44 k arrays. We used available annotations and extracted from the Agilent data gene expression values for the genes identified as significantly (P < 0.05), differentially expressed in the N1 group on our platform. Correlation coefficient of expression values of 102 identified genes was 0.925.

### Correlation of expression data with available RT-PCR and Western blot results

ATP synthase deficiency of nuclear genetic origin is characterized at the protein level by pronounced decrease of the individual subunits and the mature ATP synthase protein complex amounts (Table 2). However, our data in patient cell lines, in agreement with previous Q-PCR analyses, did not show pronounced alterations in ATP synthase subunits or of ATP synthase-specific assembly factors mRNA levels that could explain it easily. Only in the M group, the data showed decrease of *MTATP6 *and *MTATP8 *mRNA levels which correspond with previously performed Northern blot and Q-PCR analysis showing that this mutation affects processing of *ATP8/ATP6/COXIII *polycistronic transcript and results in decreased levels and/or stability of mature *ATP8/ATP6 *mRNA [19,24]. Many of mitochondrial diseases are associated with compensatory changes in the cellular content of mitochondria and/or the content of one or more OXPHOS complexes. Western blot analysis of fibroblasts with ATP synthase deficiency has previously shown increased mitochondrial content of complex I and complex III [25]. In agreement with this observation, our data showed elevated expression of complex I subunit genes in N1 group. Expression of complex III subunit genes was however decreased. Parallel analyses of the fibroblasts with nuclear ATP synthase defects used in this study revealed variable changes in fibroblast COX and/or SDH specific content (Table 2). These changes were not associated with generally elevated expression of COX and SDH subunit genes. Detailed inspection of individual gene expression profiles [see Additional file 12, 13, 14, 15, 16, 17, 18, 19, 20, 21, 22, 23, 24] however suggested that elevated expression of *COX7A2L *and *SDHA *may correlate with this observation (P3, P5, P6, P8, P10).

**Table 2:** Clinical, biochemical and molecular description of patients (P1 – P13).

Patient (group)	Phenotype	Biochemical data	Genetic defect	ATPase (% of C)	SDH (% of C)	COX (% of C)	Ref.
P1 (M)	PMR, encephalomyopathy, spastic quadruparesis, microcephalia,	lactate: 1.0–3.4 3 MGA: <15	mt9205ΔTA	*80–120	120–200	80–120	[19]
P2 (M)	transient lactic acidosis, nystagmus, GR	lactate: 3.9–10	mt9205ΔTA	*80–120	80–120	80–120	[20]
P3 (N2)	PMR, HCMP, hypotonia, peripheral neuropathy,	lactate: 1.4–10 3 MGA: 133–281	ncDNA, unknown	<30	120–200	120–200	[21]
P4 (N1)	Fatal lactic acidosis, HCMP	lactate: 30–36	ncDNA, unknown	<30	120–200	80–120	[82]
P5 (N2)	PMR, HCMP, hypotonia, dysmorphy, microcephaly	lactate: 1.6–8 3 MGA: 22–225	ncDNA, unknown	<30	>200	>200	[21]
P6 (N1)	PMR, HCMP, hypotonia, dysmorphy, microcephaly	lactate: 3.6–4.5 3 MGA: 28–260	ncDNA, unknown	<30	>200	>200	NR
P7 (N1)	PMR, HCMP, hypotonia, dysmorphy, microcephaly, epilepsy	lactate: 2.2–6.0 3 MGA: 28–161	ncDNA, unknown	<30	80–120	120–200	NR
P8 (N1)	PMR, hypotonia, dysmorphy, microcephaly	lactate: 3.6–6.7 3 MGA: 56–252	ncDNA, unknown	<30	120–200	>200	NR
P9 (N1)	PMR, hypotonia, dysmorphy, microcephaly	lactate: 2.2–10 3 MGA: 62–150	ncDNA, unknown	<30	>200	>200	[21]
P10 (N1)	PMR, hypotonia, dysmorphy, microcephaly	lactate: 1.4–4.6 3 MGA: 64–270	ncDNA, unknown	<30	120–200	120–200	NR
P11 (N2)	PMR, hypotonia, GR, HCMP dysmorphy, microcephaly	lactate: 1.5–8.2 3 MGA: 34–254	ncDNA, unknown	<10	80–120	80–120	[21]
P12 (N2)	PMR, hypotonia, HCMP	lactate: 2–6.0 3 MGA: 115–460	ncDNA, unknown	<10	80–120	80–120	[25]
P13 (N2)	PMR, GR, microcephaly, mild spasticity, hepatopathy	lactate: 1.2–3.9 3 MGA: 37–132	ncDNA, unknown	<30	120–200	120–200	[21]

Patient assignment to groups is based on DNA sequencing data (M) and results of PCA and hierarchical clustering (N1, N2). PMR – psychomotor retardation, HCMP – hypertrophic cardiomyopathy, GR – growth retardation, lactate – blood lactate (mmol/l), 3 MGA – 3-methylglutaconic aciduria (mg/g creatinine). ATPase (complex V), SDH (complex II) and COX (complex IV) represent enzyme protein content in fibroblast homogenates quantified by SDS PAGE/WB as in [19], using specific primary antibodies (MitoSciences, OR), Alexa Fluor^® ^680-labeled secondary antibodies and an Odyssey^® ^Infrared Imaging System (LI-COR Biotechnology, Lincoln, NE). Data are presented as % of control values. * Decreased content of subunit a (ATP6). NR means not reported.

## Discussion

### Platform selection and evaluation

In our work, we attempted to set up an experimental platform which will allow in a cost-effective way prospective gene expression analysis of cell lines and tissues from patients with various genetically determined OXPHOS defects. We considered availability of biological materials for the analysis, estimated the number of informative genes, evaluated gene content of commercially available microarrays and took into account instrumentation availability and platform related running costs. Since cultured skin fibroblasts are the most accessible, relatively well standardized, and multiple analysis amendable source for gene expression analysis especially in nuclear encoded OXPHOS defects, we estimated (based on Gene Expression Omnibus database data), that in fibroblasts, reliable expression signal may be obtained for approximately 6000 (HG-U95 array) to 10000 (HG-U133 Plus 2.0 Array) genes, of which only part may be meaningful to detect and understand anticipated changes in mitochondrial biology and related basic cell responses. In addition, we also evaluated representation of mitochondria encoded genes on available whole genome arrays. We found that (at the time of project planning) no complete coverage of mitochondrial tRNA, rRNA and OXPHOS structural subunits have been available on() Affymetrix HG_U95Av2 Array (contained just *TRNC*, *TRNY *and *TRNS1*), HG-U133 Plus 2.0 Array (no tRNAs, rRNAs and *ND1*, *ND4L*, *CYTB*) and Agilent 44 k Array (no tRNAs, rRNAs and *ND4L*). Considering this data and also available instrumentation, we then decided to construct focused an oligonucleotide microarray and employ competitive two-color hybridization approach with common reference experimental design.

Selected gene content allows gene expression analysis of the entire mitochondrial genome and almost all of "mitochondria" related genes in context of key DNA synthesis, growth response, regulatory and apoptotic genes. Hybridization signal was obtained from 78% of the designed oligonucleotides. Vast majority of the oligonucleotides giving no hybridization signal were designed to detect regulatory genes and transcription factor transcripts probably not transcribed in the analyzed materials. Interestingly, we detected hybridization signals for almost all mitochondrial tRNA and rRNA probes which is, in respect to oligo-dT labeling strategy, suggestive that all those transcript are also at least partially polyadenylated [26].

### Gene expression analysis in patients with defect of F_1_F_o _ATP synthase

In the work presented herein, we analyzed and compared gene expression profiles in fibroblast cell lines from 9 control individuals and 13 patients with biochemically proven but genetically heterogeneous F_1_F_o _ATP synthase deficiency. We aimed to identify gene expression changes indicating how affected cells react to and compensate for the common biochemical defect, use gene expression data to assign patients into already defined and/or putative disease subgroups, identify candidate disease causing genes, and define potential pathogenetic mechanisms associated with the disease.

The magnitude of observed expression changes was moderate with only several dozens of genes exceeding 2-fold changes. Comparing all the patient cell lines with all control cell lines, we have not identified any common and meaningful gene expression changes attributable to ATP synthase deficiency *per se*. It has been suggested recently that the degree and compartmentalization of ATP depletion may be defect specific and may thus have also specific biological consequences [27]. Our data support this view.

Cell lines with mtDNA mutation (M group) showed gene expression changes suggestive of suppressed mitochondrial biogenesis and metabolism characterized by down regulation of *TFAM *and *TFB1M*, master regulators of mitochondrial transcription, accompanied by reduced expression of other mitochondria encoded transcripts (*MTCO2*, *MTATP6*, *MTATP8*, and *MTND6*), reduced expression of *ATPAF1*, *E2F1*, *ACO2 *(component of mitochondria to nucleus retrograde pathway) and *PPARA*. This "mitochondria silencing" activity seems to be sensed and counterbalanced by elevated expression of *NRF1*, which is however not accompanied by expression changes of any NRF-1 target and/or coactivator genes [28,29]. Inhibition of mitochondrial biogenesis is synchronized with reduced expression of genes regulating the G1/S phase transition (*E2F1*, *MYC*, *Rb*, *CycA*, *CycD*, *CDK2*, *Cdc7*, *Cdc25A*, *PCNA*) and associated thymidine metabolism (*TK*, *TYMS*) [30]. We interpret this gene expression pattern as an ATP depletion mediated G1/S arrest [31] associated with synchronized replication arrest of mitochondrial genome [32] and repression of NRF-1 activity [33]. Our observations are quite similar to that made in *Drosophila *mutants, in which low ATP levels lead to arrest in the G1 phase without affecting cellular differentiation and cell viability [34,35]. Furthermore, our observation conforms to the view that mitochondria co-regulate cell cycle progression and that this regulation is executed not only at posttranscriptional [34] but also at transcriptional level.

The N1 group differed from M group in that it showed very minor signs of mitochondrial response suggested only by slightly elevated expression of *PPGC-1*, *TFAM*, *TFB2M *and *ACO2*. More significant and distinct changes were however observed in signal transduction pathways regulating mitochondrial oxidative phosphorylation [36]. The gene expression portrait, reduced expression of many transcription factors and cytokines regulating cell growth and differentiation (*FOS *[37], *JUNB *and *MAPK3 *[38], *CEBPA *and *CEBPB*, *CXCL1 *and *CXCL2 *[39]), elevated expression of *IGFBP3 *[40] and *CAV2 *[41], together with activated apoptosis (*BCL2L1*, *SMAC*, *CYCS*, *FAF1*), signs of oxidative stress (*TR2*) [42] and general decrease in lysosomal activities [43], resemble characteristic signs of senescent fibroblasts [44,45]. However all the cell lines from the N1 group have originated from very young donors, all but one were in their early passages and all showed the same passage frequency of 5–6 days, (Table 3). It has been shown that inhibition of oxidative phosphorylation may play an active role in the process of cellular senescence in human fibroblasts [46], and that changes in transcription activity may be governed by changes in protein phosphorylation [47]. We therefore interpret the observed gene expression pattern as accelerated stress induced premature senescence phenotype resulting from impaired oxidative phosphorylation and profoundly reduced ATP availability for critical energy-dependent cellular processes. Our explanation of N1 cellular phenotype is the following. Mitochondrial ATP synthesis is markedly decreased in fibroblasts derived from patients with nuclear DNA-related disorders but only variably so in patients with mtDNA mutations [48]. ATP depletion is sensed by AMP-activated protein kinase which acts as a metabolic sensor or "fuel gauge" that monitors cellular AMP and ATP levels [49]. Once activated, the enzyme switches off ATP-consuming anabolic pathways and switches on ATP-producing catabolic pathways [50], such as fatty acid oxidation (elevated expression of *ECH1, ECHS1, ETFDH, CABC1*) and amino acid catabolism. Despite this compensatory effort, mitochondrial ATP depletion persists due to intrinsic ATP synthase defect, activation of AMPK persist and leads to accelerated p53-dependent cellular senescence [51]. AMPK activity also leads to decrease of HuR cytosolic translocation, which influences the mRNA-stabilizing function of HuR [52] and diminishes the expression and half-lives of HuR target transcripts, such as *FOS *[53] or *CDKN1A *[54] which also leads to the premature senescence phenotype [55]. ATP availability probably modulates cytoplasmic translocation and recruitment of other RNA-binding proteins stabilizing various mRNAs [56]. In this context it is interesting that we have detected reduced expression (or transcript abundance) of two RNA-binding protein genes *CUGBP1 *and *AUH*. CUGBP1 affects translation of *CDKN1A *[57] and *CEBPB *[58], and our data show decrease in those two transcripts as well. AUH stabilizes *FOS *and other immediate early mRNA's [59], and its deficiency is also causing methylglutaconic aciduria [60], a characteristic biochemical phenotype observed specifically in this group of nuclear encoded ATP synthase deficient patients [21] (Table 2).

**Table 3:** Growth characteristics of the fibroblast cell lines.

	**patients**													**controls**								
	1	2	3	4	5	6	7	8	9	10	11	12	13	1	2	3	4	5	6	7	8	9
passage number	17	19	15	4	20	9	6	6	28	12	28	17	12	22	22	14	27	16	17	11	16	13
passage frequency (days)	5	5	6	6	6	6	6	6	5	6	6	7	7	9	3	3	4	4	5	4	4	7

Resulting transcriptional silencing and other ATP depletion mediated disturbances of intracellular signal transduction cascades lead thus to premature senescence phenotype, reduced proteasome activity and accumulation of oxidized proteins, which may explain observed discrepancies between gene expression and Western blot data. Patients forming this group are of common ethnic origin, and this is suggestive that common genetic defect may underlie this specific gene expression profile.

The N2 group showed neither signs of mitochondria response observed in the M group, nor signs of premature senescence observed in the N1 group. Expression profile is suggestive of partly activated apoptosis (*SMAC*) and disturbances of intracellular signaling transduction cascades (down regulation of several cytokines, early genes, and regulatory proteins). However, all these changes were not uniformly present in all cell lines, which together with variability in clinical and biochemical data is suggestive of further genetic heterogeneity within this group of patients.

### Selection of candidate disease causing genes

As gene expression changes may be used for selection of candidate disease causing genes [9,61], we evaluated group specific and individual gene expression profiles. This approach was successful in both patients from the M group, in whom detected alterations clearly indicated the involvement of ATP6/ATP8/COXIII transcript. In other patients we first focused on expression of ATP synthase subunits. Inspection of this expression profile (Figure 5B) suggested involvement of ATP synthase assembly factor ATPAF2 in several patients from N1 group. Mutation of *ATPAF2 *has been found in the case with ATP synthase deficiency [62] and this warrant sequence analysis of this gene in this group of patients. Other candidate genes may be *ATP5G2*, the expression of which is decreased in P5 and possibly also *ATP5C1 *found lowered in P3. From other genes, no clear candidates for immediate sequence analysis may be defined yet. However, more focused interpretation will be possible once candidate disease genomic intervals are defined by ongoing linkage studies.

## Conclusion

We designed, produced, and validated an oligonucleotide microarray focused on expression profiling of human mitochondria related genes, and searched for gene expression changes in genetically heterogeneous group of 13 patients with F_1_F_o _ATP synthase deficiency. The analysis classified patients into three distinct groups and suggested that site (mtDNA vs nucleus) and severity (residual content of ATP synthase) of underlying biochemical defect have diverse effects on cell gene expression phenotype. Comparisons with controls, between defined groups and among individual patient cell lines did not show any uniform transcription changes explaining pronounced decrease in ATP synthase content and alterations of the other OXPHOS complexes observed at the protein level. The analysis nevertheless confirmed the already known and indicated candidate disease causing genes, and suggested that defects in ATP synthesis lead to deregulation of signal transduction pathways and affect mitochondrial and nuclear DNA replication. These may be important pathogenic mechanisms involved not only in F_1_F_o _ATP synthase deficiency but also in other OXPHOS defects. Observed gene expression changes therefore warrant further investigation of major cell cycle regulatory and signal transduction pathways in other OXPHOS disorders and pharmacological models. Full potential of the constructed h-MitoArray platform will be further revealed in ongoing positional cloning studies in herein analyzed patients and in gene expression studies in other groups of OXPHOS deficient cell lines.

## Methods

### Database of human mitochondrial genes

Lists of "mitochondrial" and "mitochondria related" genes were extracted and merged from various public databases such as Mitomap [63], Mitop [64], Migenes [65], Mitoproteom [66], Molecular Signature Database [67], OMIM, RefSeq and Unigene sections at NCBI [68], Gene Ontology database [69] and UniProt resource [70]. Full annotation of selected genes has been obtained and deposited in a locally installed database BASE [71].

### Microarray preparation

For each of the selected 1632 genes, a single 5'-aminomodified 40-mer oligonucleotide was designed using Oligopicker software [72]. Blast searches were performed with each candidate probe to exclude possibility of cross hybridization with homologous genes prior to the synthesis of oligonucleotide probes. Synthesized oligonucleotides, Generi Biotech (Czech Republic) and Illumina (San Diego, CA), were resuspended at 20 μM concentration in 3 × SSC, printed in triplicates on aminosilane modified slides, and immobilised by standard technique using combination of baking and UV cross-link as previously described [61]. Qualities of arrays from individual printing series were assessed using fluorescently labelled panomers (Invitrogen, Carlsbad, CA).

### Mixed RNA for microarray validation

As a standard for microarray optimisation, standardization and validation total RNA was isolated from HeLa G, ECV 304, 293, U 937, JURKAT and A 301 cell lines using the TRIZOL solution (Invitrogen, Carlsbad, CA). Isolated RNA samples were pooled, and aliquots were stored at -80°C until the analysis.

### Reference RNA preparation

As a common reference RNA for gene expression studies, total RNA from cultured HeLa cells was chosen. Total RNA was extracted as above. Concentration was determined spectrophotometrically at A 260 by NanoDrop (NanoDrop Technologies, Wilmington, DE) and quality was checked on Agilent 2100 bioanalyser – RNA Lab-On-a-Chip (Agilent Technologies, Santa Clara, CA). Aliquots of isolated RNA were stored at -80°C until the analysis.

### Control group

Selected control fibroblasts cell lines were used repeatedly in previous diagnostic biochemical tests and showed no signs of any mitochondrial or other metabolic defect.

### Patients

Fibroblast cell lines from 13 patients were used in this study. All the patients showed major clinical symptoms associated with OXPHOS defect. Biochemical diagnosis of ATP synthase deficiency was based on absence or significant decrease of mature ATP synthase complex and of its subunits in electrophoretic analysis of OXPHOS complexes in cultured fibroblasts and other available tissues [73]. Mitochondrial genome sequencing performed in all patients revealed disease causing mitochondrial DNA mutations in two patients (P1, P2, M group) [19]. Molecular basis of defect in the other patients has not yet been defined. Relevant clinical, biochemical and molecular data and references on individual patients included in this study are provided in Table 2.

### Cell culturing

Growth characteristics of the cell lines used in this study are provided in Table 3. Skin fibroblasts were cultured in the Dulbecco's modified Eagle's medium supplemented by 10% fetal calf serum, 20 mM HEPES pH 7.5, 0.2% NaHCO_3 _and gentamycin 0.02 mg/ml at 37°C in a 5% CO_2 _humidified atmosphere. For experiments, confluent cell were harvested using 0.05% trypsine and 0.02% EDTA. Detached cells were diluted in ice-cold culture medium, sedimented by centrifugation (600 g) and washed twice in phosphate buffered saline (140 mM NaCl, 5.4 mM KCl, 8 mM Na_2_HPO_4_, 1.4 mM KH_2_PO_4_, pH 7.2).

### RNA preparation, cDNA labeling and hybridization

Total RNA was extracted from cultured cells and QC controlled as described above.

Five μg of total RNA was reverse transcribed and labeled by Array 900 Expression Detection Kit (Genisphere, Hatfield, PA) according to the manufacturer protocol. The slides were pretreated by baking at 80°C, UV cross-linked and washed twice in 0.1% SDS for 2 minutes, twice in 0.2 × SSC for 2 min, four times in MilliQ water, followed by denaturation in boiling water for 2 minutes. Prehybridization was performed using hybridization buffer (Genisphere, Hatfield, PA) according to the manufacturer protocol. All hybridizations were performed in humid hybridization chamber, ArrayIt Hybridization Cassette chamber (TeleChem International, Sunnyvale, CA).

### Microarray scanning

The hybridized slides were scanned with GenePix 4200A scanner (Axon Instruments, Union City, CA) with PMT gains adjusted to obtain highest intensity unsaturated images. GenePix Pro software (Axon Instruments, Union City, CA) was used for image analysis of the TIFF files, as generated by the scanner.

### Experimental setup and data normalization

All 13 patient samples and 9 controls were hybridized to common reference (HeLa cell lines) in two replicates of each sample. All arrays were hybridized with a Cy5-labeled sample cDNA and a Cy3-labeled reference cDNA.

Expression data were obtained using GenPix Pro software. Comparative microarray analysis was performed according to MIAME guidelines [74]. Normalization was performed in R statistical environment [75] using Limma package [76] which is part of the Bioconductor project [77]. Raw data from individual arrays were processed using Loess normalization and normexp background correction. Gquantile function was used for normalization between arrays. The correlation between 3 replicate spots per gene on each array was used to increase the robustness. Linear model was fitted for each gene given a series of arrays using lmFit function. The empirical Bayes method was used to rank differential expression of genes using eBayes function. Multiple testing correction was performed using Benjamini & Hochberg method [78].

### Quality control

Variation among feature replicates on the array was calculated by conversion of raw data to log-ratios. Data were further normalized using Loess function. Features with less than double background intensity (A < 8.5) were removed. For each feature on the array the deviation from the mean computed as the difference between the ratio of the feature and the mean of the set of feature replicates was calculated. Standard deviation of the error distribution using all of the replicates was calculated and converted to coefficient of variability using equation.

$$\text{CV}=\sqrt{\exp[{\left(\ln2*\text{SD}2\right)}^{2}]-1}$$

The variability between the duplicate spots ranged from 8.1% to 27.5%. Arrays with variability higher then 18% were removed from the analysis.

### Statistical analysis

Principal component analysis, hierarchical clustering, ANOVA and SAM analyses were performed in TIGR Multiexperiment Viewer (MeV), version 4.0 [79], available [80]. Significant gene expression changes between defined subgroups were identified using t-test in R statistical environment [75]. Applied parameters are provided in corresponding result sections.

### Functional annotation

Functional annotation and pathway enrichment analysis was performed in DAVID (The Database for Annotation, Visualization and Integrated Discovery [23]). Visualization of gene expression changes along affected pathways was performed in KEGGArray software (KEGG pathway databases – Kyoto Encyclopedia of Genes and Genomes) [81].

### Data accession

Description of h-MitoArray platform and gene expression data reported in this study are stored and available in Gene Expression Omnibus repository under accessions GPL5150 and GSE8648.

### Ethics

The project was approved by the Scientific Ethics Committee of the 1st Faculty of Medicine of Charles University of Prague under reference NR/8069-3. Patient participation in the project was made on a voluntary basis after oral and written information and consent according to the Helsinki V Declaration.

## Authors' contributions

A.Č. tested, compared and optimized labeling and hybridization condition, performed all the RNA sample isolations, cDNA labeling, microarray hybridizations, data acquisition, and contributed to data analysis and interpretation. V.S performed all data analysis in R and MeV environments. R.I. designed oligonucleotide probes, updated functional annotation of selected gene set, and participated in data analysis. H.H., L.N. a L.P. set and kept BASE database, optimized methods for microarray manufacturing and prepared all microarrays used in this study. M.T, H. Han., T.H., J.Z., J.A.M., W.S., A.P., J.P. and J.H. performed all relevant clinical, biochemical, and molecular investigations in studied patients, and together with S.S. provided patient and control cell lines. S.K. conceived and coordinated the study, was involved together with J.H. in data analysis, result interpretation and manuscript preparation.

## Supplementary Material

Additional file 1 Annotation of h-MitoArray. List of selected genes with corresponding symbols, accession and LocusLink codes. Probes showing hybridization signal with fluorescently labeled panomers and fluorescently labeled cDNA are presented as "signal detected". Click here for file

Additional file 2 Functional annotation of the h-MitoArray. Functional annotation of selected genes and comparison of the h-MitoArray gene content against whole human genome reference set. Click here for file

Additional file 3 Expression matrix – samples compared to common reference. Ratios of Log2 sample gene intensities against Log2 gene intensities of common reference. Click here for file

Additional file 4 Expression matrix – patients compared to controls. Ratios of individual patient Log2 gene intensities against the average of the Log2 controls intensities. Click here for file

Additional file 5 Differentially expressed genes between all patients and controls. List of genes detected as differentially expressed between all studied ATP synthase deficient and control fibroblast cell lines at adjusted P < 0.01 significance level. Click here for file

Additional file 6 Characterization of defined patient groups using ANOVA analysis. List of genes detected as differentially expressed between defined patient groups using ANOVA analysis and unadjusted P < 0.01 significance level. Click here for file

Additional file 7 Lists of differentially expressed genes in M group. Lists of genes detected as differentially expressed in M group, when compared to controls at adjusted P < 0.01 significance level. Click here for file

Additional file 8 Lists of differentially expressed genes in N1 group. Lists of genes detected as differentially expressed in N1 group, when compared to controls at adjusted P < 0.01 significance level. Click here for file

Additional file 9 Lists of differentially expressed genes in N2 group. Lists of genes detected as differentially expressed in N2 group, when compared to controls at adjusted P < 0.01 significance level. Click here for file

Additional file 10 Pathway analysis in M group. Pathways and gene expression changes identified by KEGGArray software in M group. Click here for file

Additional file 11 Pathway analysis in N1 group. Pathways and gene expression changes identified by KEGGArray software in N1 group. Click here for file

Additional file 12 Differentially expressed genes in patient P1. Lists of genes detected as differentially expressed in individual patients comparing to controls at adjusted P < 0.01 significance level. Click here for file

Additional file 13 Differentially expressed genes in patient P2. Lists of genes detected as differentially expressed in individual patients comparing to controls at adjusted P < 0.01 significance level. Click here for file

Additional file 14 Differentially expressed genes in patient P3. Lists of genes detected as differentially expressed in individual patients comparing to controls at adjusted P < 0.01 significance level. Click here for file

Additional file 15 Differentially expressed genes in patient P4. Lists of genes detected as differentially expressed in individual patients comparing to controls at adjusted P < 0.01 significance level. Click here for file

Additional file 16 Differentially expressed genes in patient P5. Lists of genes detected as differentially expressed in individual patients comparing to controls at adjusted P < 0.01 significance level. Click here for file

Additional file 17 Differentially expressed genes in patient P6. Lists of genes detected as differentially expressed in individual patients comparing to controls at adjusted P < 0.01 significance level. Click here for file

Additional file 18 Differentially expressed genes in patient P7. Lists of genes detected as differentially expressed in individual patients comparing to controls at adjusted P < 0.01 significance level. Click here for file

Additional file 19 Differentially expressed genes in patient P8. Lists of genes detected as differentially expressed in individual patients comparing to controls at adjusted P < 0.01 significance level. Click here for file

Additional file 20 Differentially expressed genes in patient P9. Lists of genes detected as differentially expressed in individual patients comparing to controls at adjusted P < 0.01 significance level. Click here for file

Additional file 21 Differentially expressed genes in patient P10. Lists of genes detected as differentially expressed in individual patients comparing to controls at adjusted P < 0.01 significance level. Click here for file

Additional file 22 Differentially expressed genes in patient P11. Lists of genes detected as differentially expressed in individual patients comparing to controls at adjusted P < 0.01 significance level. Click here for file

Additional file 23 Differentially expressed genes in patient P12. Lists of genes detected as differentially expressed in individual patients comparing to controls at adjusted P < 0.01 significance level. Click here for file

Additional file 24 Differentially expressed genes in patient P13. Lists of genes detected as differentially expressed in individual patients comparing to controls at adjusted P < 0.01 significance level. Click here for file
