# Supplementary material for: Development of a human mitochondrial oligonucleotide microarray (h-MitoArray) and gene expression analysis of fibroblast cell lines from 13 patients with isolated F1Fo ATP synthase deficiency
Source: BMC Genomics. 2008 Jan 25;9:38. doi: 10.1186/1471-2164-9-38 (PMC2267714; doi:10.1186/1471-2164-9-38)
Supplement: Additional file 10 — Pathway analysis in M group. Pathways and gene expression changes identified by KEGGArray software in M group. [file 1471-2164-9-38-S10.PDF]

M group

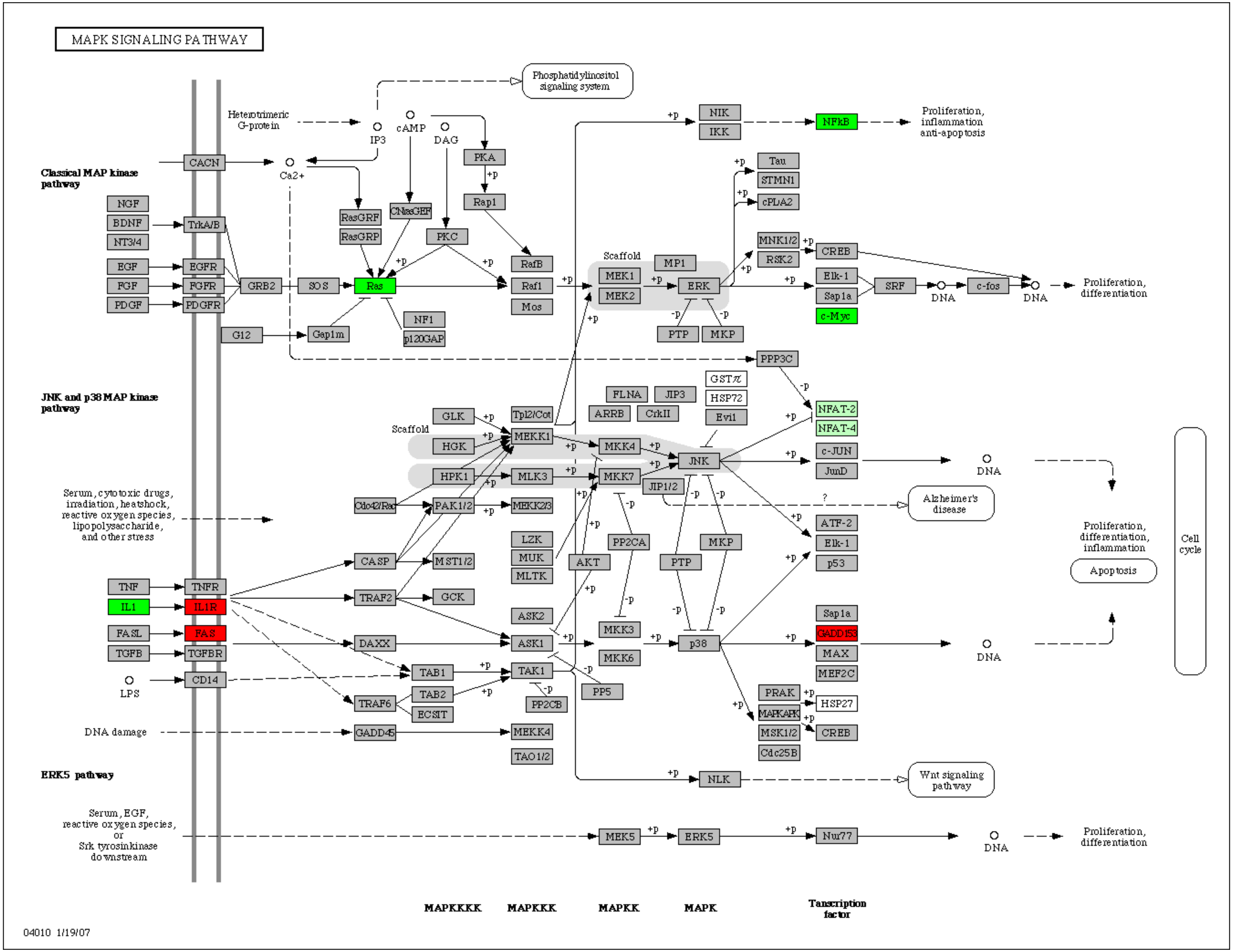
MAPK SIGNALING PATHWAY
Classical MAP kinase pathway
JNK and p38 MAP kinase pathway
ERK5 pathway
MAPKKK
MAPKK
MAPK
MAPK
Tanscription factor
04010 1/19/07

M group

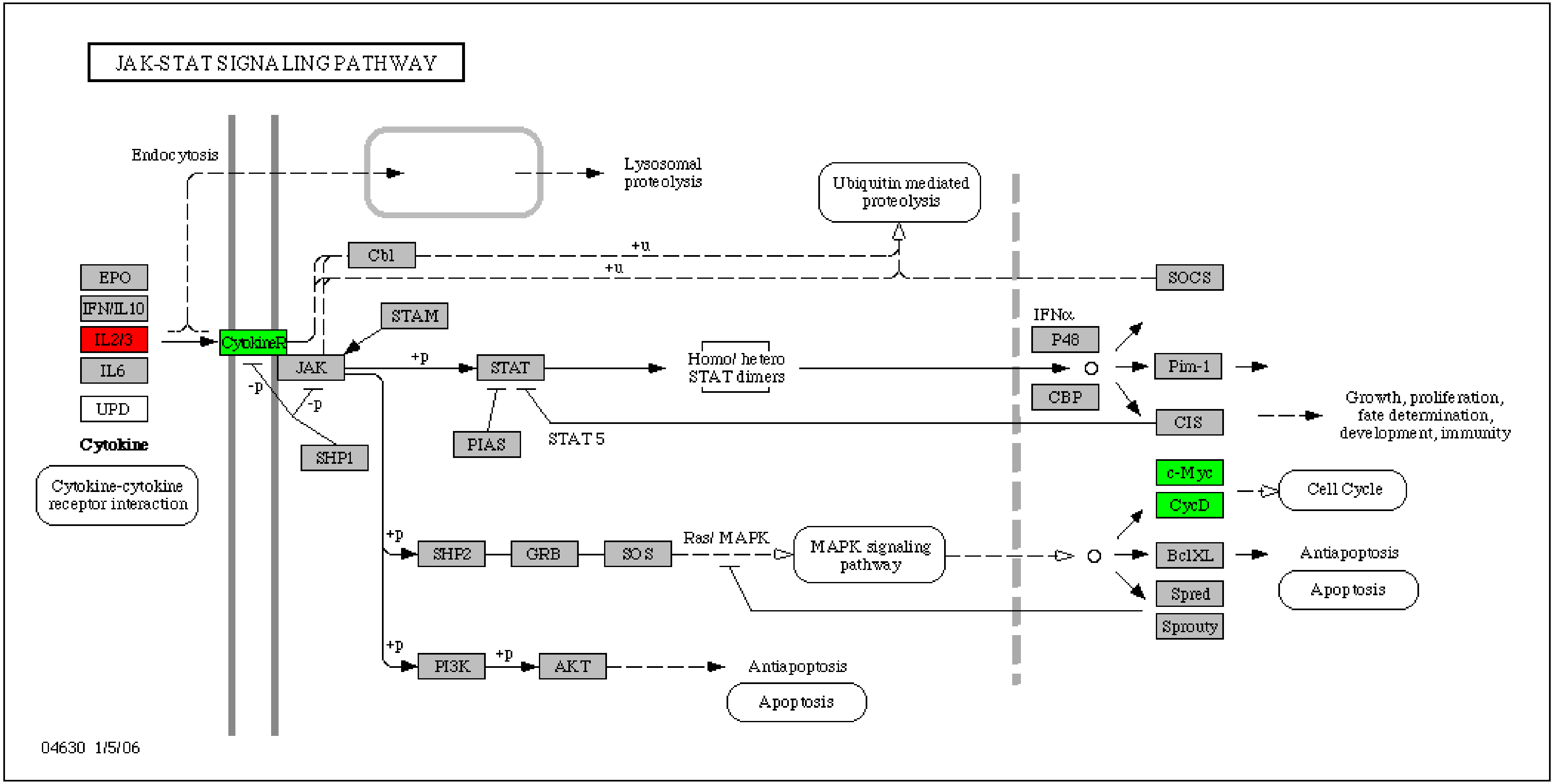
JAK-STAT SIGNALING PATHWAY
Endocytosis
Lysosomal proteolysis
Ubiquitin mediated proteolysis
Cbl
+u
+u
SOCS
EPO
IFN/IL10
IL2/3
IL6
UPD
Cytokine
Cytokine-cytokine receptor interaction
CytokineR
STAM
JAK
-p
-p
SHP1
+p
STAT
PIAS
STAT5
Homo/ hetero STAT dimers
IFNα
P48
CBP
Pim-1
CIS
Growth, proliferation, fate determination, development, immunity
c-Myc
CycD
Cell Cycle
+p
SHP2
GRB
SOS
Ras/ MAPK
MAPK signaling pathway
BclXL
Antiapoptosis
Apoptosis
Spred
Sprouty
+p
PI3K
+p
AKT
Antiapoptosis
Apoptosis
04630 1/5/06

M group

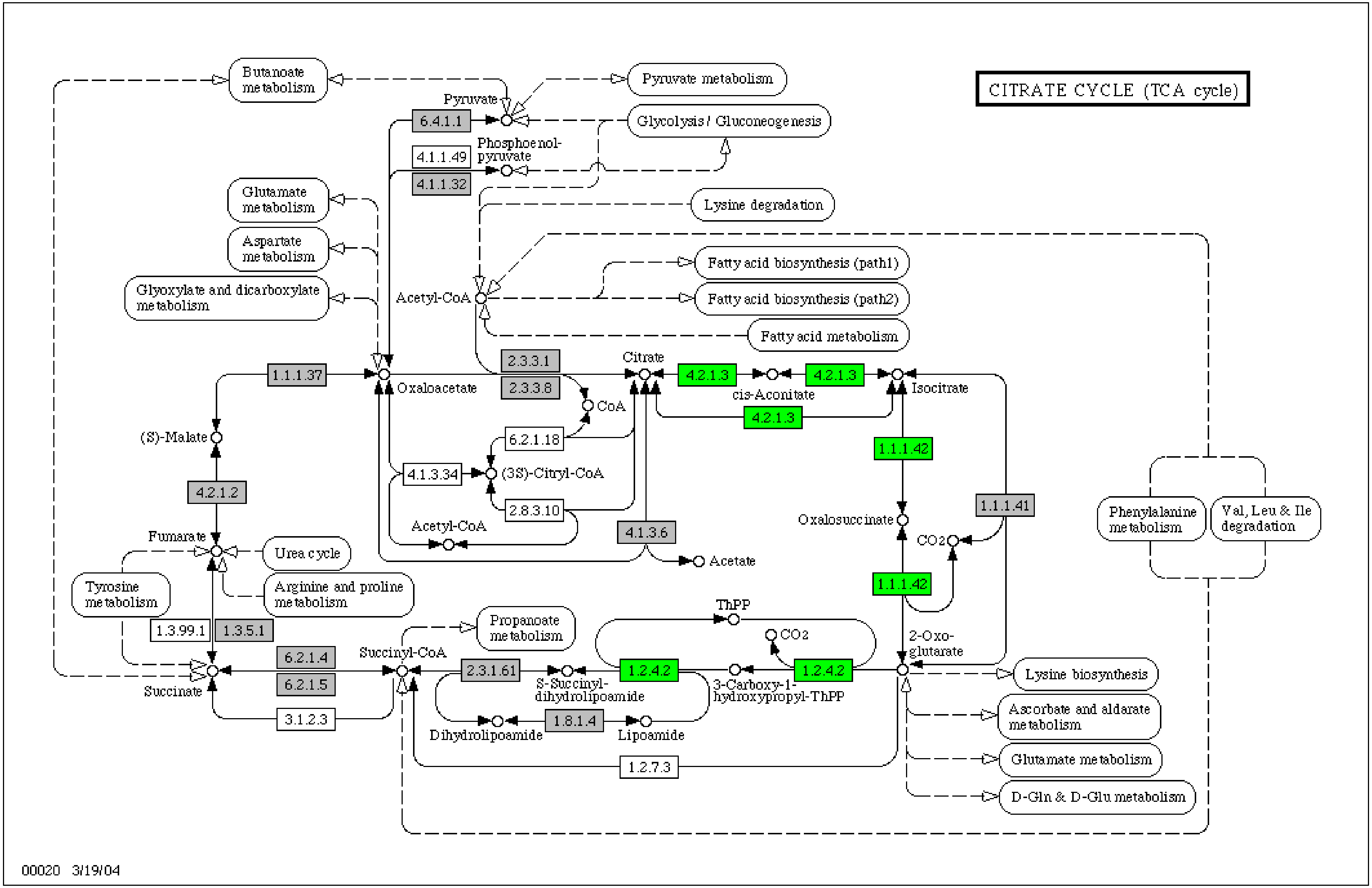
CITRATE CYCLE (TCA cycle)
Butanoate metabolism
Pyruvate metabolism
Pyruvate
Glycolysis / Gluconeogenesis
6.4.1.1
4.1.1.49
4.1.1.32
Phosphoenol-pyruvate
Glutamate metabolism
Lysine degradation
Aspartate metabolism
Fatty acid biosynthesis (path1)
Glyoxylate and dicarboxylate metabolism
Acetyl-CoA
Fatty acid biosynthesis (path2)
Fatty acid metabolism
2.3.3.1
2.3.3.8
Citrate
4.2.1.3
4.2.1.3
1.1.1.37
Oxaloacetate
cis-Aconitate
Isocitrate
CoA
4.2.1.3
(S)-Malate
6.2.1.18
1.1.1.42
4.1.3.34
(3S)-Citryl-CoA
4.2.1.2
2.8.3.10
1.1.1.41
Phenylalanine metabolism
Val, Leu & Ile degradation
Acetyl-CoA
4.1.3.6
Oxalosuccinate
Fumarate
CO2
Urea cycle
Acetate
Tyrosine metabolism
Arginine and proline metabolism
1.1.1.42
ThPP
Propanoate metabolism
CO2
1.3.99.1
1.3.5.1
2-Oxo-glutarate
6.2.1.4
Succinyl-CoA
2.3.1.61
1.2.4.2
1.2.4.2
6.2.1.5
S-Succinyl-dihydrolipoamide
3-Carboxy-1-hydroxypropyl-ThPP
Lysine biosynthesis
Succinate
3.1.2.3
1.8.1.4
Ascorbate and aldarate metabolism
Dihydrolipoamide
Lipoamide
1.2.7.3
Glutamate metabolism
D-Gln & D-Glu metabolism
00020 3/19/04

M group

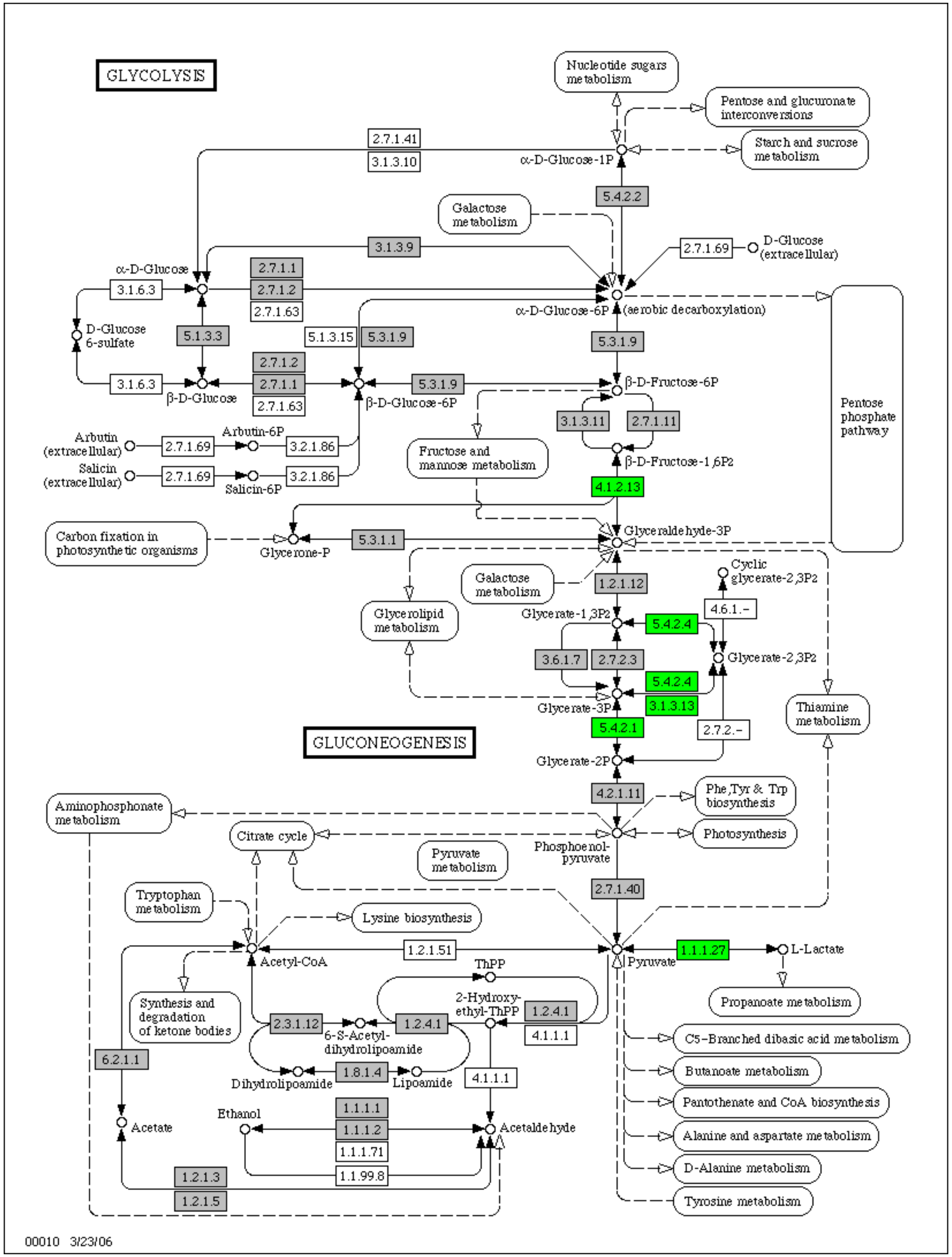
GLYCOLYSIS
Nucleotide sugars metabolism
Pentose and glucuronate interconversions
Starch and sucrose metabolism
2.7.1.41
3.1.3.10
α-D-Glucose-1P
5.4.2.2
Galactose metabolism
3.1.3.9
2.7.1.69
D-Glucose (extracellular)
α-D-Glucose
2.7.1.1
2.7.1.2
2.7.1.63
3.1.6.3
D-Glucose 6-sulfate
5.1.3.3
α-D-Glucose-6P
(aerobic decarboxylation)
5.1.3.15
5.3.1.9
2.7.1.2
2.7.1.1
2.7.1.63
5.3.1.9
3.1.6.3
β-D-Glucose
β-D-Glucose-6P
β-D-Fructose-6P
3.1.3.11
2.7.1.11
Arbutin (extracellular)
2.7.1.69
Arbutin-6P
3.2.1.86
Salicin (extracellular)
2.7.1.69
Salicin-6P
3.2.1.86
Fructose and mannose metabolism
β-D-Fructose-1,6P2
4.1.2.13
Pentose phosphate pathway
Carbon fixation in photosynthetic organisms
Glycerone-P
5.3.1.1
Glyceraldehyde-3P
Galactose metabolism
1.2.1.12
Cyclic glycerate-2,3P2
4.6.1.-
Glycerolipid metabolism
Glycerate-1,3P2
5.4.2.4
3.6.1.7
2.7.2.3
Glycerate-2,3P2
5.4.2.4
3.1.3.13
Glycerate-3P
5.4.2.1
2.7.2.-
Thiamine metabolism
GLUCONEOGENESIS
Glycerate-2P
4.2.1.11
Phe,Tyr & Trp biosynthesis
Aminophosphonate metabolism
Citrate cycle
Phosphoenol-pyruvate
Photosynthesis
Pyruvate metabolism
2.7.1.40
Tryptophan metabolism
Lysine biosynthesis
Acetyl-CoA
1.2.1.51
Pyruvate
1.1.1.27
L-Lactate
ThPP
Propanoate metabolism
Synthesis and degradation of ketone bodies
2-Hydroxy-ethyl-ThPP
1.2.4.1
2.3.1.12
1.2.4.1
4.1.1.1
6-S-Acetyl-dihydrolipoamide
C5-Branched dibasic acid metabolism
6.2.1.1
1.8.1.4
Dihydrolipoamide
Lipoamide
4.1.1.1
Butanoate metabolism
Pantothenate and CoA biosynthesis
Ethanol
1.1.1.1
1.1.1.2
Acetate
Acetaldehyde
Alanine and aspartate metabolism
1.1.1.71
1.1.99.8
D-Alanine metabolism
1.2.1.3
1.2.1.5
Tyrosine metabolism
00010 3/23/06

M group

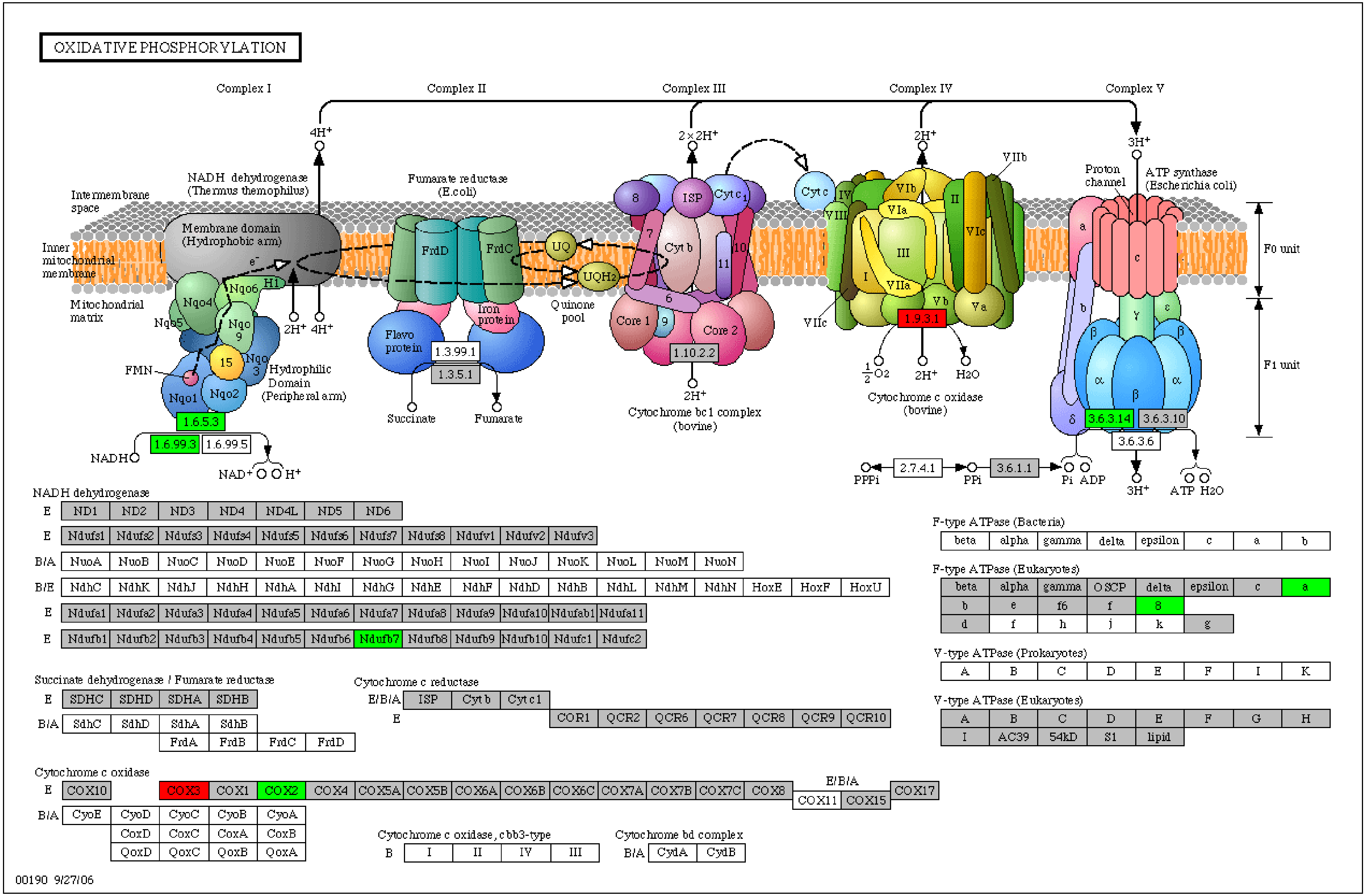
OXIDATIVE PHOSPHORYLATION
Complex I
Complex II
Complex III
Complex IV
Complex V
4H+
2×2H+
2H+
3H+
Intermembrane space
Inner mitochondrial membrane
Mitochondrial matrix
NADH dehydrogenase (Thermus themophilus)
Membrane domain (Hydrophobic arm)
Hydrophilic Domain (Peripheral arm)
Nqo4
Nqo5
Nqo6
H1
Nqo9
15
Nqo3
Nqo1
Nqo2
FMN
e-
2H+
4H+
1.6.5.3
NADH
1.6.99.3
1.6.99.5
NAD+
H+
Fumarate reductase (E.coli)
FrdD
FrdC
Flavo protein
Iron protein
1.3.99.1
1.3.5.1
Succinate
Fumarate
UQ
UQH2
Quinone pool
8
ISP
Cyt c1
Cyt c
7
Cyt b
10
11
6
9
Core 1
Core 2
1.10.2.2
Cytochrome bc1 complex (bovine)
VIIb
IV
VIII
VIb
VIa
II
VIc
III
I
VIIa
Vb
Va
VIIc
1.9.3.1
1/2 O2
H2O
Cytochrome c oxidase (bovine)
Proton channel
ATP synthase (Escherichia coli)
a
b
c
γ
ε
β
α
δ
F0 unit
F1 unit
3.6.3.14
3.6.3.10
3.6.3.6
PPPi
2.7.4.1
PPi
3.6.1.1
Pi
ADP
ATP
NADH dehydrogenase
E ND1 ND2 ND3 ND4 ND4L ND5 ND6
E Ndufs1 Ndufs2 Ndufs3 Ndufs4 Ndufs5 Ndufs6 Ndufs7 Ndufs8 Ndufv1 Ndufv2 Ndufv3
B/A NuoA NuoB NuoC NuoD NuoE NuoF NuoG NuoH NuoI NuoJ NuoK NuoL NuoM NuoN
B/E NdhC NdhK NdhJ NdhH NdhA NdhI NdhG NdhE NdhF NdhD NdhB NdhL NdhM NdhN HoxE HoxF HoxU
E Ndufa1 Ndufa2 Ndufa3 Ndufa4 Ndufa5 Ndufa6 Ndufa7 Ndufa8 Ndufa9 Ndufa10 Ndufab1 Ndufa11
E Ndufb1 Ndufb2 Ndufb3 Ndufb4 Ndufb5 Ndufb6 Ndufb7 Ndufb8 Ndufb9 Ndufb10 Ndufc1 Ndufc2
Succinate dehydrogenase / Fumarate reductase
E SDHC SDHD SDHA SDHB
B/A SdhC SdhD SdhA SdhB
FrdA FrdB FrdC FrdD
Cytochrome c reductase
E/B/A ISP Cyt b Cyt c1
E COR1 QCR2 QCR6 QCR7 QCR8 QCR9 QCR10
Cytochrome c oxidase
E COX10 COX3 COX1 COX2 COX4 COX5A COX5B COX6A COX6B COX6C COX7A COX7B COX7C COX8
E/B/A COX11 COX15
COX17
B/A CyoE CyoD CyoC CyoB CyoA
CoxD CoxC CoxA CoxB
QoxD QoxC QoxB QoxA
Cytochrome c oxidase, cbb3-type
B I II IV III
Cytochrome bd complex
B/A CydA CydB
F-type ATPase (Bacteria)
beta alpha gamma delta epsilon c a b
F-type ATPase (Eukaryotes)
beta alpha gamma OSCP delta epsilon c a
b e f6 f 8
d f h j k g
V-type ATPase (Prokaryotes)
A B C D E F I K
V-type ATPase (Eukaryotes)
A B C D E F G H
I AC39 54kD S1 lipid
00190 9/27/06

M group

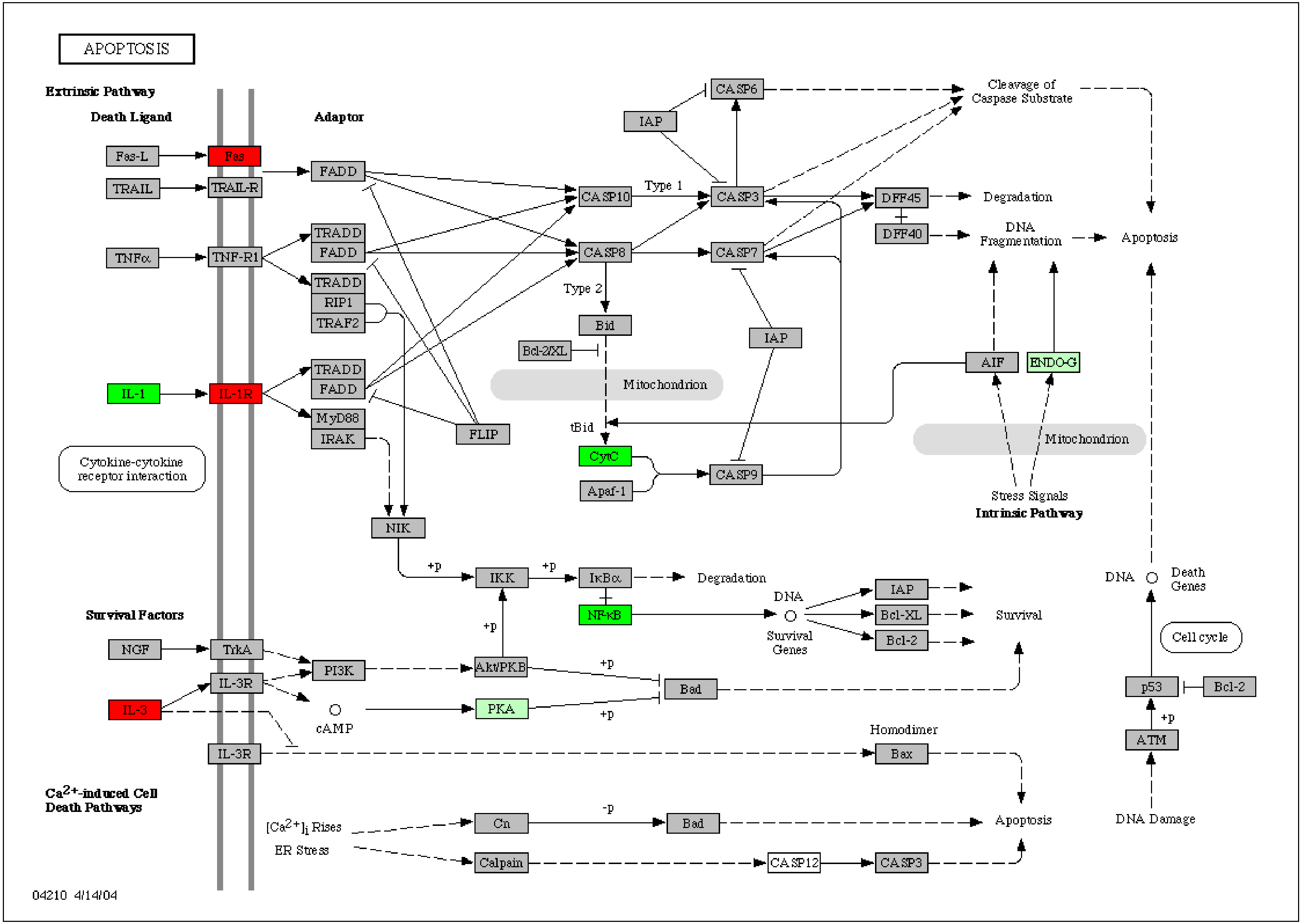
APOPTOSIS
Extrinsic Pathway
Death Ligand
Adaptor
Fas-L
Fas
TRAIL
TRAIL-R
TNFα
TNF-R1
IL-1
IL-1R
FADD
TRADD
FADD
TRADD
RIP1
TRAF2
TRADD
FADD
MyD88
IRAK
FLIP
Cytokine-cytokine receptor interaction
NIK
IAP
CASP6
Cleavage of Caspase Substrate
CASP10
Type 1
CASP3
DFF45
Degradation
DFF40
DNA Fragmentation
Apoptosis
CASP8
CASP7
Type 2
Bid
Bcl-2/XL
IAP
Mitochondrion
tBid
CytC
Apaf-1
CASP9
AIF
ENDO-G
Mitochondrion
Stress Signals
Intrinsic Pathway
+p
IKK
+p
IκBα
Degradation
NF-κB
DNA
Survival Genes
IAP
Bcl-XL
Bcl-2
Survival
DNA
Death Genes
Cell cycle
p53
Bcl-2
+p
ATM
DNA Damage
Survival Factors
NGF
TrkA
PI3K
+p
Akt/PKB
+p
Bad
IL-3R
IL-3
cAMP
PKA
+p
IL-3R
Homodimer
Bax
Ca2+-induced Cell Death Pathways
[Ca2+]i Rises
ER Stress
Cn
-p
Bad
Apoptosis
Calpain
CASP12
CASP3
04210 4/14/04

M group

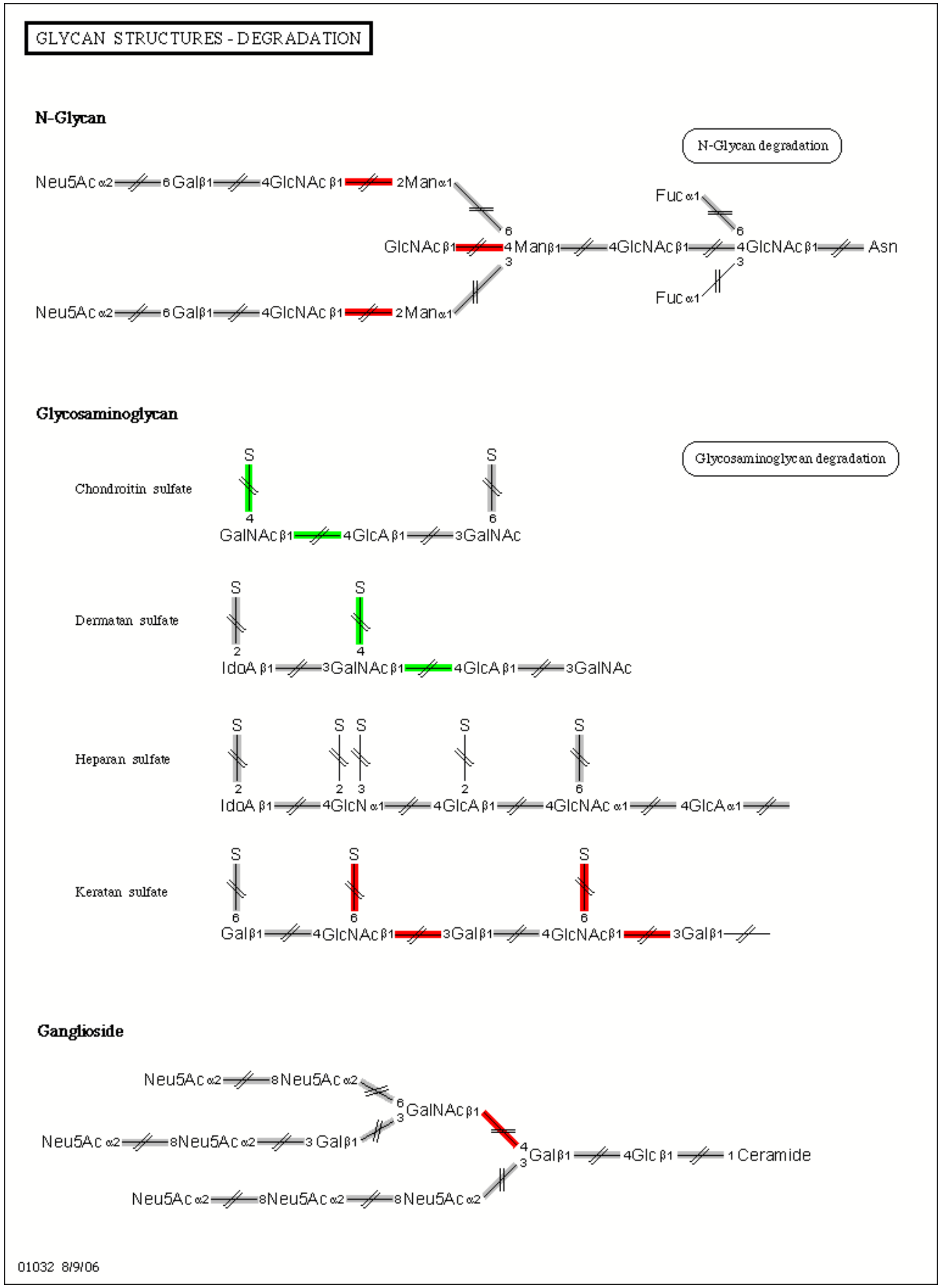
GLYCAN STRUCTURES - DEGRADATION
N-Glycan
N-Glycan degradation
Neu5Acα2 6Galβ1 4GlcNAcβ1 2Manα1
Fucα1
GlcNAcβ1 4Manβ1 4GlcNAcβ1 4GlcNAcβ1 Asn
Fucα1
Neu5Acα2 6Galβ1 4GlcNAcβ1 2Manα1
Glycosaminoglycan
Glycosaminoglycan degradation
Chondroitin sulfate
GalNAcβ1 4GlcAβ1 3GalNAc
Dermatan sulfate
IdoAβ1 3GalNAcβ1 4GlcAβ1 3GalNAc
Heparan sulfate
IdoAβ1 4GlcNα1 4GlcAβ1 4GlcNAcα1 4GlcAα1
Keratan sulfate
Galβ1 4GlcNAcβ1 3Galβ1 4GlcNAcβ1 3Galβ1
Ganglioside
Neu5Acα2 8Neu5Acα2
GalNAcβ1
Neu5Acα2 8Neu5Acα2 3Galβ1
Galβ1 4Glcβ1 1Ceramide
Neu5Acα2 8Neu5Acα2 8Neu5Acα2
01032 8/9/06

M group

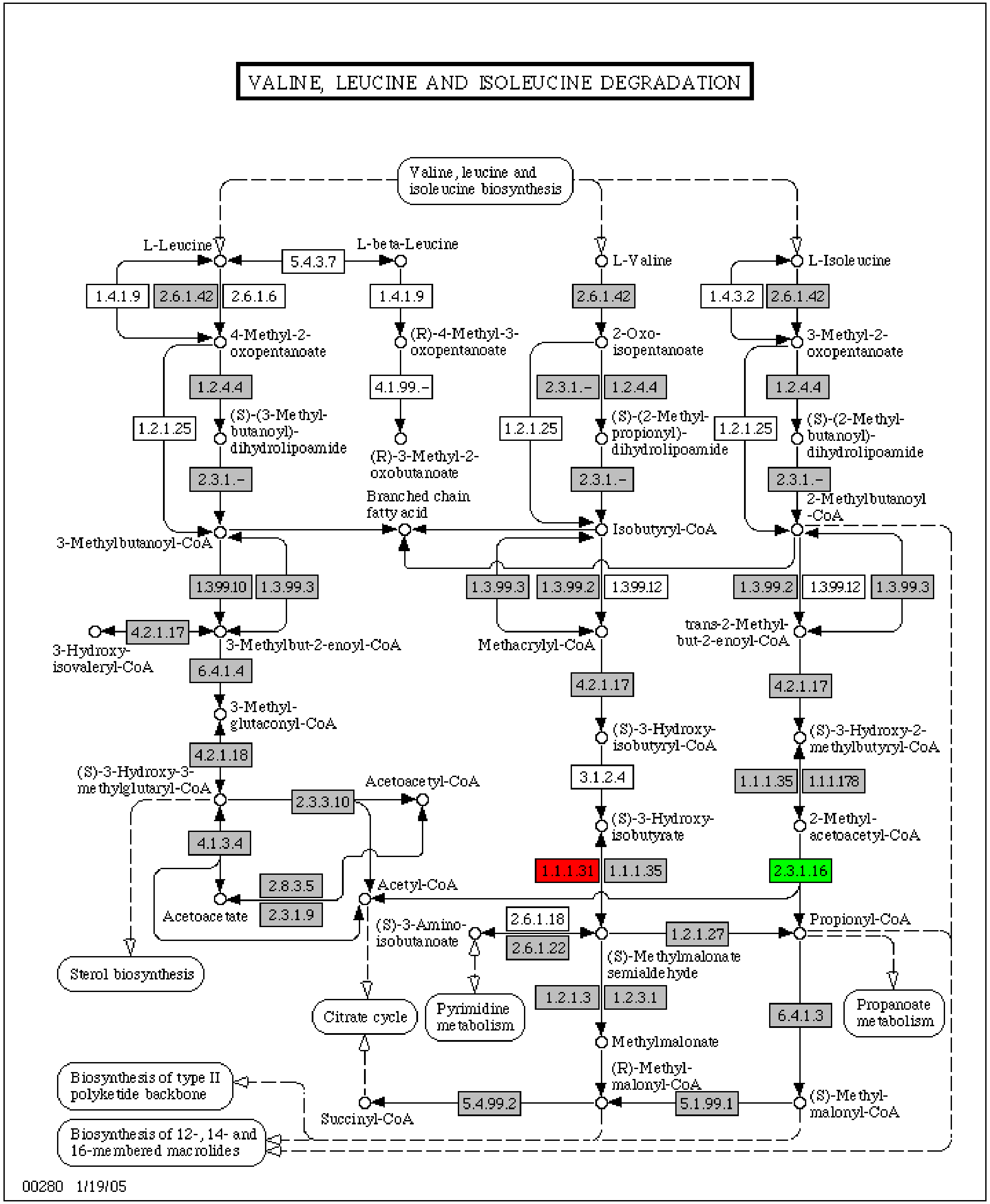
VALINE, LEUCINE AND ISOLEUCINE DEGRADATION
Valine, leucine and isoleucine biosynthesis
L-Leucine
L-beta-Leucine
L-Valine
L-Isoleucine
5.4.3.7
1.4.1.9
2.6.1.42
2.6.1.6
1.4.1.9
2.6.1.42
1.4.3.2
2.6.1.42
4-Methyl-2-oxopentanoate
(R)-4-Methyl-3-oxopentanoate
2-Oxo-isopentanoate
3-Methyl-2-oxopentanoate
1.2.4.4
4.1.99.-
2.3.1.-
1.2.4.4
1.2.4.4
1.2.1.25
(S)-(3-Methyl-butanoyl)-dihydrolipoamide
1.2.1.25
(S)-(2-Methyl-propionyl)-dihydrolipoamide
1.2.1.25
(S)-(2-Methyl-butanoyl)-dihydrolipoamide
(R)-3-Methyl-2-oxobutanoate
2.3.1.-
2.3.1.-
2.3.1.-
Branched chain fatty acid
2-Methylbutanoyl-CoA
3-Methylbutanoyl-CoA
Isobutyryl-CoA
1.3.99.10
1.3.99.3
1.3.99.3
1.3.99.2
1.3.99.12
1.3.99.2
1.3.99.12
1.3.99.3
4.2.1.17
3-Methylbut-2-enoyl-CoA
Methacrylyl-CoA
trans-2-Methyl-but-2-enoyl-CoA
3-Hydroxy-isovaleryl-CoA
6.4.1.4
4.2.1.17
4.2.1.17
3-Methyl-glutaconyl-CoA
(S)-3-Hydroxy-isobutyryl-CoA
(S)-3-Hydroxy-2-methylbutyryl-CoA
4.2.1.18
3.1.2.4
1.1.1.35
1.1.1.178
(S)-3-Hydroxy-3-methylglutaryl-CoA
2.3.3.10
Acetoacetyl-CoA
(S)-3-Hydroxy-isobutyrate
2-Methyl-acetoacetyl-CoA
4.1.3.4
1.1.1.31
1.1.1.35
2.3.1.16
2.8.3.5
Acetyl-CoA
Acetoacetate
2.3.1.9
(S)-3-Amino-isobutanoate
2.6.1.18
2.6.1.22
1.2.1.27
Propionyl-CoA
Sterol biosynthesis
(S)-Methylmalonate semialdehyde
1.2.1.3
1.2.3.1
Citrate cycle
Pyrimidine metabolism
6.4.1.3
Propanoate metabolism
Methylmalonate
(R)-Methyl-malonyl-CoA
Biosynthesis of type II polyketide backbone
5.4.99.2
5.1.99.1
(S)-Methyl-malonyl-CoA
Succinyl-CoA
Biosynthesis of 12-, 14- and 16-membered macrolides
00280 1/19/05

M group

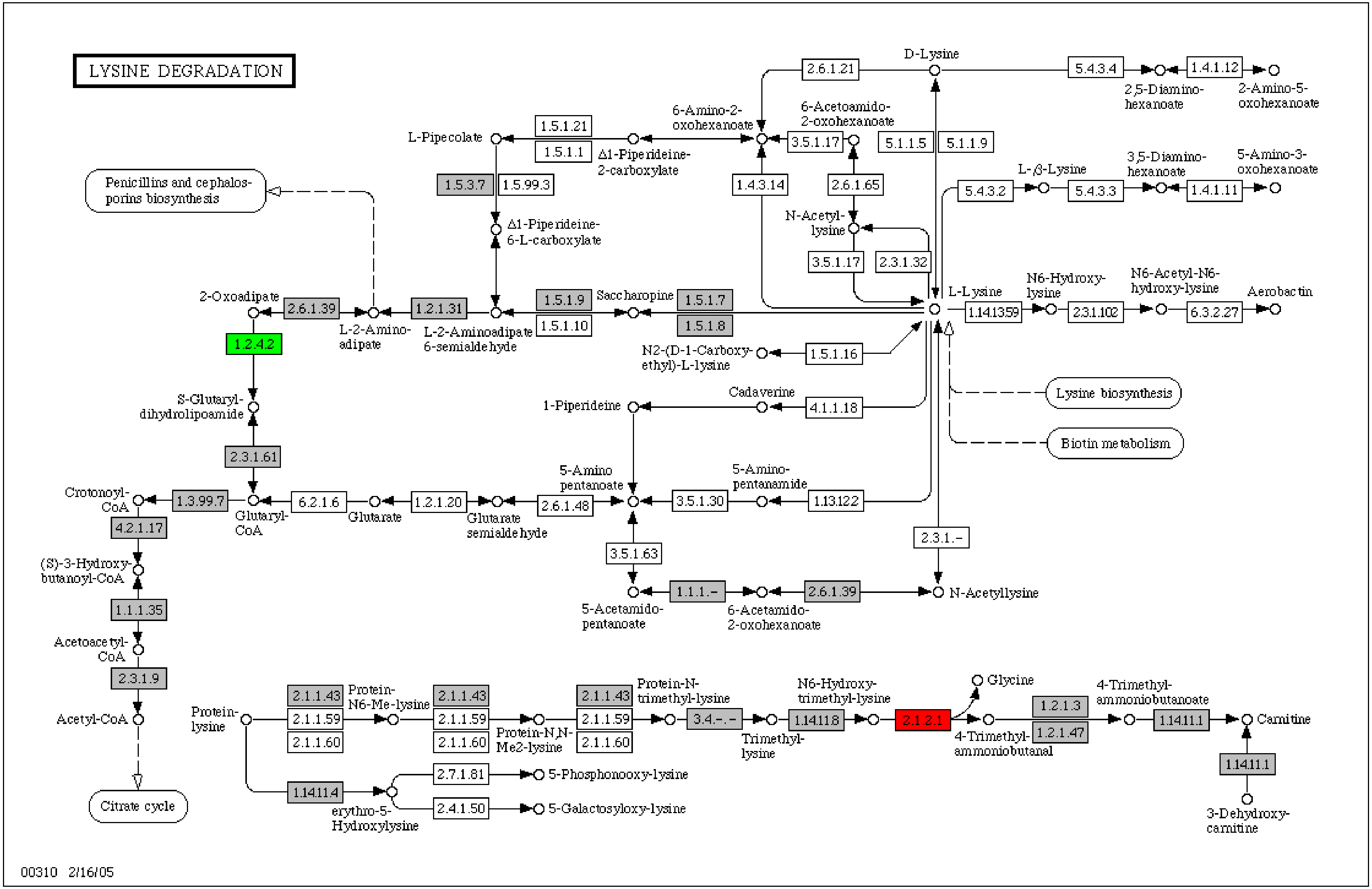

LYSINE DEGRADATION
Penicillins and cephalosporins biosynthesis
L-Pipecolate
1.5.1.21
1.5.1.1
Δ1-Piperideine-2-carboxylate
6-Amino-2-oxohexanoate
6-Acetoamido-2-oxohexanoate
3.5.1.17
5.1.1.5
5.1.1.9
D-Lysine
2.6.1.21
5.4.3.4
1.4.1.12
2,5-Diaminohexanoate
2-Amino-5-oxohexanoate
1.5.3.7
1.5.99.3
1.4.3.14
2.6.1.65
5.4.3.2
L-β-Lysine
5.4.3.3
3,5-Diaminohexanoate
1.4.1.11
5-Amino-3-oxohexanoate
Δ1-Piperideine-6-L-carboxylate
N-Acetyl-lysine
3.5.1.17
2.3.1.32
2-Oxoadipate
2.6.1.39
1.2.1.31
1.5.1.9
Saccharopine
1.5.1.7
1.5.1.8
1.5.1.10
L-2-Aminoadipate
L-2-Aminoadipate 6-semialdehyde
L-Lysine
1.14.13.59
N6-Hydroxy-lysine
2.3.1.102
N6-Acetyl-N6-hydroxy-lysine
6.3.2.27
Aerobactin
1.2.4.2
N2-(D-1-Carboxyethyl)-L-lysine
1.5.1.16
S-Glutaryl-dihydrolipoamide
1-Piperideine
Cadaverine
4.1.1.18
Lysine biosynthesis
Biotin metabolism
2.3.1.61
5-Amino pentanoate
5-Amino-pentanamide
Crotonoyl-CoA
1.3.99.7
Glutaryl-CoA
6.2.1.6
Glutarate
1.2.1.20
Glutarate semialdehyde
2.6.1.48
3.5.1.30
1.13.12.2
4.2.1.17
2.3.1.-
(S)-3-Hydroxy-butanoyl-CoA
3.5.1.63
1.1.1.35
1.1.1.-
2.6.1.39
N-Acetyllysine
5-Acetamido-pentanoate
6-Acetamido-2-oxohexanoate
Acetoacetyl-CoA
2.3.1.9
Acetyl-CoA
Protein-lysine
2.1.1.43
2.1.1.59
2.1.1.60
Protein-N6-Me-lysine
Protein-N,N-Me2-lysine
Protein-N-trimethyl-lysine
3.4.-.-
Trimethyl-lysine
N6-Hydroxy-trimethyl-lysine
1.14.11.8
2.1.2.1
Glycine
4-Trimethyl-ammoniobutanal
1.2.1.3
1.2.1.47
4-Trimethyl-ammoniobutanoate
1.14.11.1
Carnitine
3-Dehydroxy-carnitine
Citrate cycle
1.14.11.4
2.7.1.81
5-Phosphonooxy-lysine
2.4.1.50
5-Galactosyloxy-lysine
erythro-5-Hydroxylysine
00310 2/16/05

M group

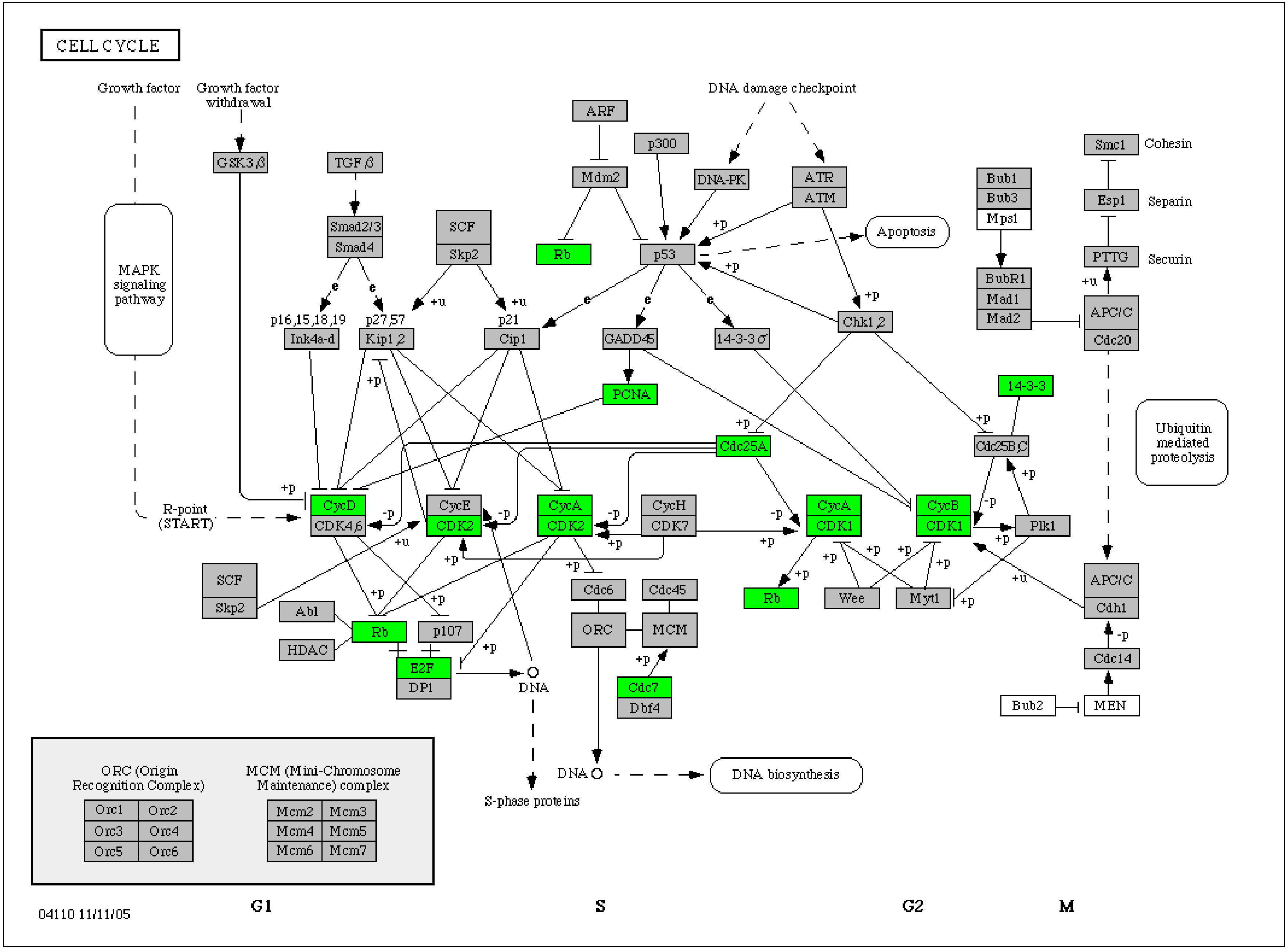
CELL CYCLE
Growth factor
Growth factor withdrawal
DNA damage checkpoint
GSK3β
TGFβ
ARF
Mdm2
p300
DNA-PK
ATR
ATM
Smc1
Cohesin
Bub1
Bub3
Mps1
Esp1
Separin
MAPK signaling pathway
Smad2/3
Smad4
SCF
Skp2
Rb
p53
Apoptosis
PTTG
Securin
BubR1
Mad1
Mad2
APC/C
Cdc20
p16,15,18,19
Ink4a-d
p27,57
Kip1,2
p21
Cip1
GADD45
14-3-3σ
Chk1,2
PCNA
14-3-3
Cdc25A
Cdc25B,C
Ubiquitin mediated proteolysis
R-point (START)
CycD
CDK4,6
CycE
CDK2
CycA
CDK2
CycH
CDK7
CycA
CDK1
CycB
CDK1
Plk1
SCF
Skp2
Abl
HDAC
Rb
p107
E2F
DP1
DNA
Cdc6
Cdc45
ORC
MCM
Cdc7
Dbf4
Rb
Wee
Myt1
APC/C
Cdh1
Cdc14
Bub2
MEN
DNA
DNA biosynthesis
S-phase proteins
ORC (Origin Recognition Complex)
Orc1
Orc2
Orc3
Orc4
Orc5
Orc6
MCM (Mini-Chromosome Maintenance) complex
Mcm2
Mcm3
Mcm4
Mcm5
Mcm6
Mcm7
04110 11/11/05
G1
S
G2
M

M group

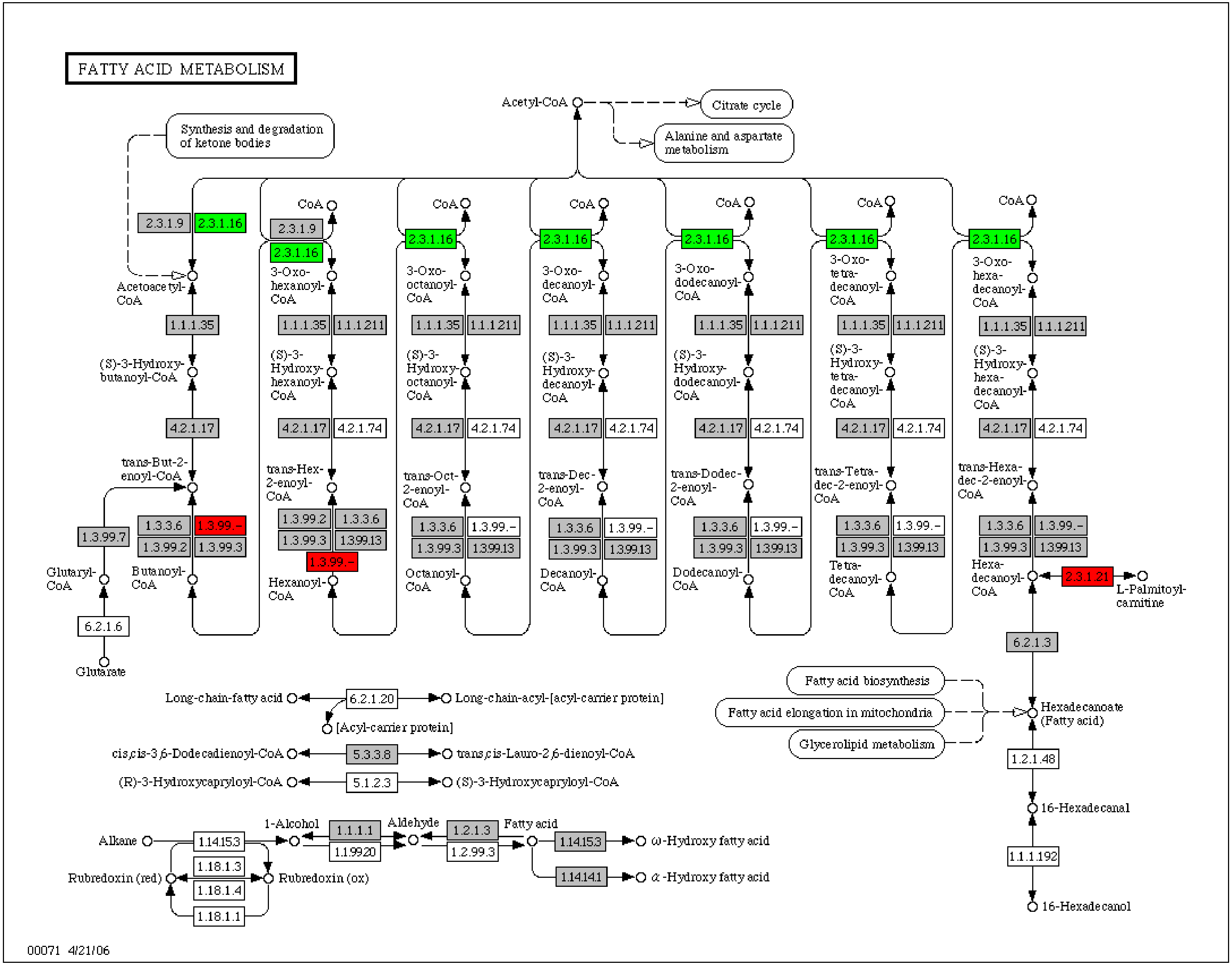
FATTY ACID METABOLISM
Acetyl-CoA
Citrate cycle
Alanine and aspartate metabolism
Synthesis and degradation of ketone bodies
2.3.1.9
2.3.1.16
CoA
Acetoacetyl-CoA
3-Oxo-hexanoyl-CoA
3-Oxo-octanoyl-CoA
3-Oxo-decanoyl-CoA
3-Oxo-dodecanoyl-CoA
3-Oxo-tetra-decanoyl-CoA
3-Oxo-hexa-decanoyl-CoA
1.1.1.35
1.1.1.211
(S)-3-Hydroxy-butanoyl-CoA
(S)-3-Hydroxy-hexanoyl-CoA
(S)-3-Hydroxy-octanoyl-CoA
(S)-3-Hydroxy-decanoyl-CoA
(S)-3-Hydroxy-dodecanoyl-CoA
(S)-3-Hydroxy-tetra-decanoyl-CoA
(S)-3-Hydroxy-hexa-decanoyl-CoA
4.2.1.17
4.2.1.74
trans-But-2-enoyl-CoA
trans-Hex-2-enoyl-CoA
trans-Oct-2-enoyl-CoA
trans-Dec-2-enoyl-CoA
trans-Dodec-2-enoyl-CoA
trans-Tetra-dec-2-enoyl-CoA
trans-Hexa-dec-2-enoyl-CoA
1.3.99.7
1.3.3.6
1.3.99.-
1.3.99.2
1.3.99.3
1.3.99.13
Glutaryl-CoA
Butanoyl-CoA
Hexanoyl-CoA
Octanoyl-CoA
Decanoyl-CoA
Dodecanoyl-CoA
Tetra-decanoyl-CoA
Hexa-decanoyl-CoA
2.3.1.21
L-Palmitoyl-carnitine
6.2.1.6
Glutarate
6.2.1.3
Fatty acid biosynthesis
Fatty acid elongation in mitochondria
Glycerolipid metabolism
Hexadecanoate (Fatty acid)
1.2.1.48
16-Hexadecanal
1.1.1.192
16-Hexadecanol
Long-chain-fatty acid
6.2.1.20
Long-chain-acyl-[acyl-carrier protein]
[Acyl-carrier protein]
cis,cis-3,6-Dodecadienoyl-CoA
5.3.3.8
trans,cis-Lauro-2,6-dienoyl-CoA
(R)-3-Hydroxycapryloyl-CoA
5.1.2.3
(S)-3-Hydroxycapryloyl-CoA
Alkane
1.14.15.3
1.18.1.3
1.18.1.4
1.18.1.1
Rubredoxin (red)
Rubredoxin (ox)
1-Alcohol
1.1.1.1
1.1.99.20
Aldehyde
1.2.1.3
1.2.99.3
Fatty acid
1.14.15.3
ω-Hydroxy fatty acid
1.14.14.1
α-Hydroxy fatty acid
00071 4/21/06
