# Supplementary material for: Development of a human mitochondrial oligonucleotide microarray (h-MitoArray) and gene expression analysis of fibroblast cell lines from 13 patients with isolated F1Fo ATP synthase deficiency
Source: BMC Genomics. 2008 Jan 25;9:38. doi: 10.1186/1471-2164-9-38 (PMC2267714; doi:10.1186/1471-2164-9-38)
Supplement: Additional file 11 — Pathway analysis in N1 group. Pathways and gene expression changes identified by KEGGArray software in N1 group. [file 1471-2164-9-38-S11.PDF]

N1 group

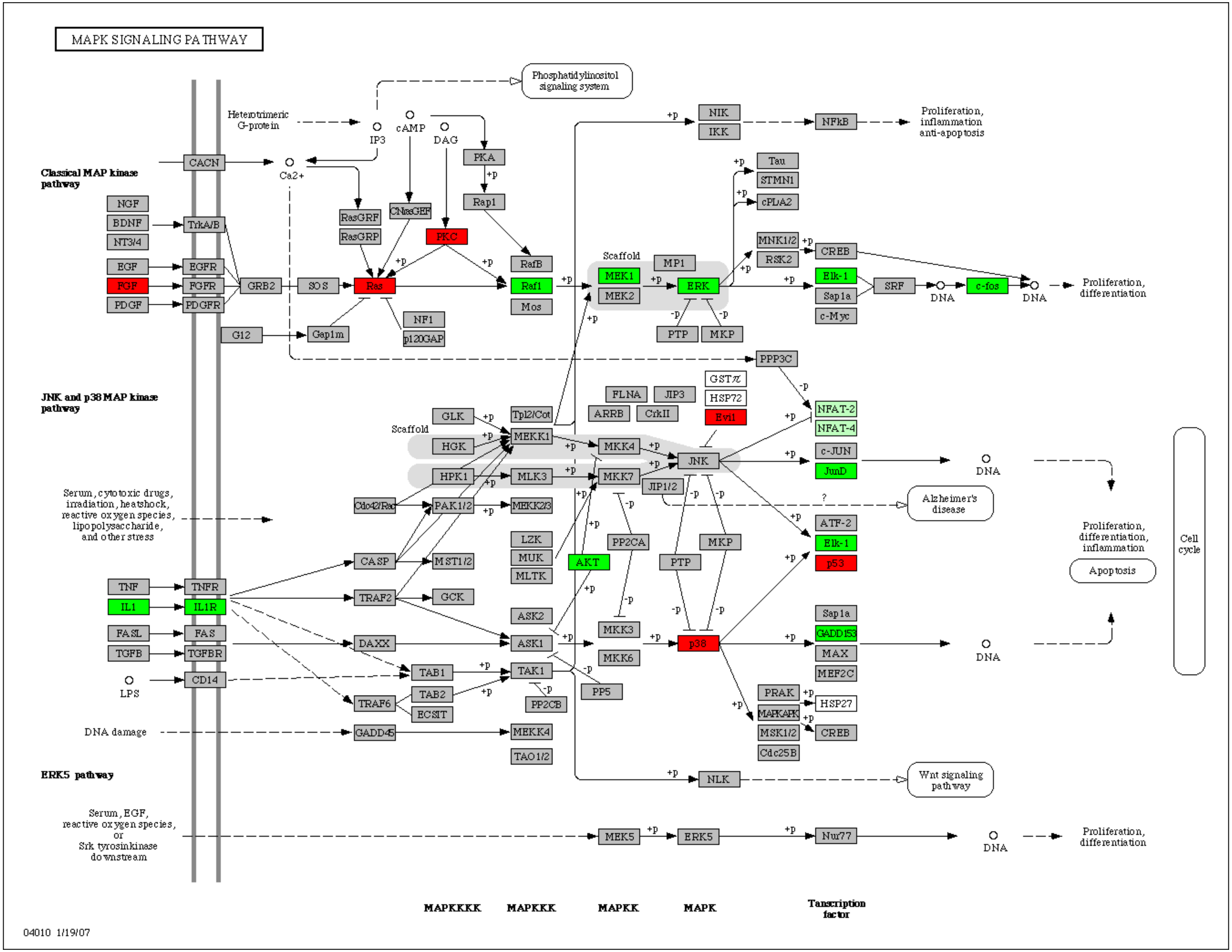

MAPK SIGNALING PATHWAY
Phosphatidylinositol signaling system
Heterotrimeric G-protein
IP3
cAMP
DAG
CACN
Ca2+
PKA
Rap1
Classical MAP kinase pathway
NGF
BDNF
NT3/4
TrkA/B
EGF
FGF
PDGF
EGFR
FGFR
PDGFR
GRB2
SOS
Ras
RasGRF
RasGRP
CNrasGEF
PKC
RafB
Raf1
Mos
G12
Gap1m
NF1
p120GAP
NIK
IKK
NFkB
Proliferation, inflammation anti-apoptosis
Tau
STMN1
cPLA2
MNK1/2
RSK2
CREB
Scaffold
MP1
MEK1
MEK2
ERK
PTP
MKP
Elk-1
Sap1a
c-Myc
SRF
c-fos
DNA
Proliferation, differentiation
PPP3C
JNK and p38 MAP kinase pathway
GLK
HGK
HPK1
Tpl2/Cot
MEKK1
MLK3
Cdc42/Rac
PAK1/2
MEKK2/3
FLNA
JIP3
ARRB
CrkII
GSTπ
HSP72
Evi1
MKK4
MKK7
JIP1/2
JNK
NFAT-2
NFAT-4
c-JUN
JunD
Alzheimer's disease
Serum, cytotoxic drugs, irradiation, heatshock, reactive oxygen species, lipopolysaccharide, and other stress
LZK
MUK
MLTK
AKT
PP2CA
MKP
ATF-2
Elk-1
p53
Proliferation, differentiation, inflammation
Apoptosis
Cell cycle
TNF
IL1
FASL
TGFB
LPS
TNFR
IL1R
FAS
TGFBR
CD14
CASP
TRAF2
DAXX
TAB1
TAB2
TRAF6
ECSIT
MST1/2
GCK
ASK2
ASK1
TAK1
PP2CB
PP5
MKK3
MKK6
p38
Sap1a
GADD153
MAX
MEF2C
PRAK
MAPKAPK
MSK1/2
Cdc25B
HSP27
CREB
DNA damage
GADD45
MEKK4
TAO1/2
NLK
Wnt signaling pathway
ERK5 pathway
Serum, EGF, reactive oxygen species, or Srk tyrosinkinase downstream
MEK5
ERK5
Nur77
MAPKKK
MAPKK
MAPKK
MAPK
Tanscription factor
04010 1/19/07

N1 group

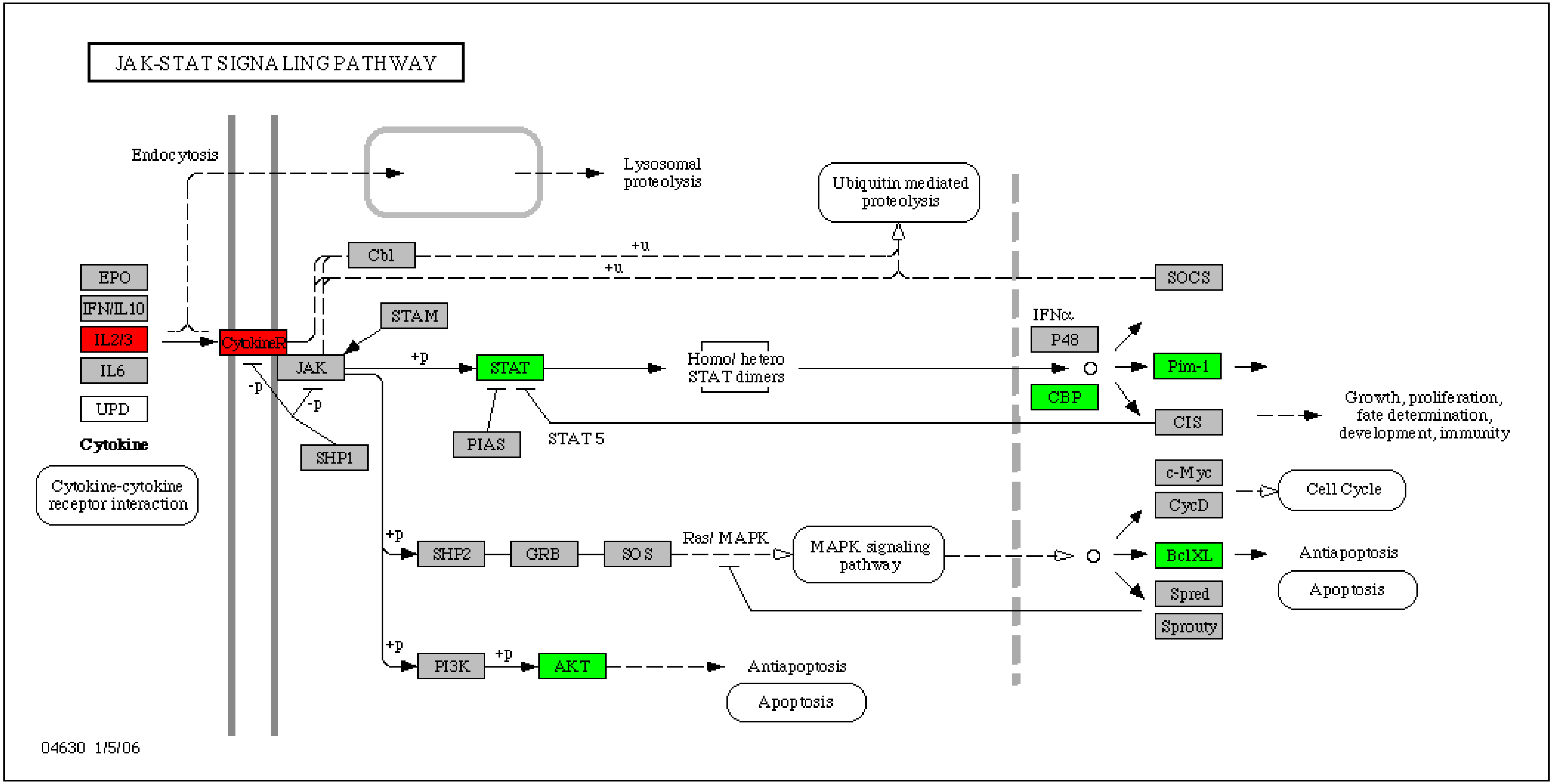

JAK-STAT SIGNALING PATHWAY
Endocytosis
Lysosomal proteolysis
Ubiquitin mediated proteolysis
Cbl
+u
+u
SOCS
EPO
IFN/IL10
IL2/3
IL6
UPD
Cytokine
Cytokine-cytokine receptor interaction
CytokineR
STAM
JAK
-p
-p
SHP1
+p
STAT
PIAS
STAT5
Homo/ hetero STAT dimers
IFNα
P48
CBP
Pim-1
CIS
Growth, proliferation, fate determination, development, immunity
c-Myc
CycD
Cell Cycle
+p
SHP2
GRB
SOS
Ras/ MAPK
MAPK signaling pathway
BclXL
Antiapoptosis
Apoptosis
Spred
Sprouty
+p
PI3K
+p
AKT
Antiapoptosis
Apoptosis
04630 1/5/06

N1 group

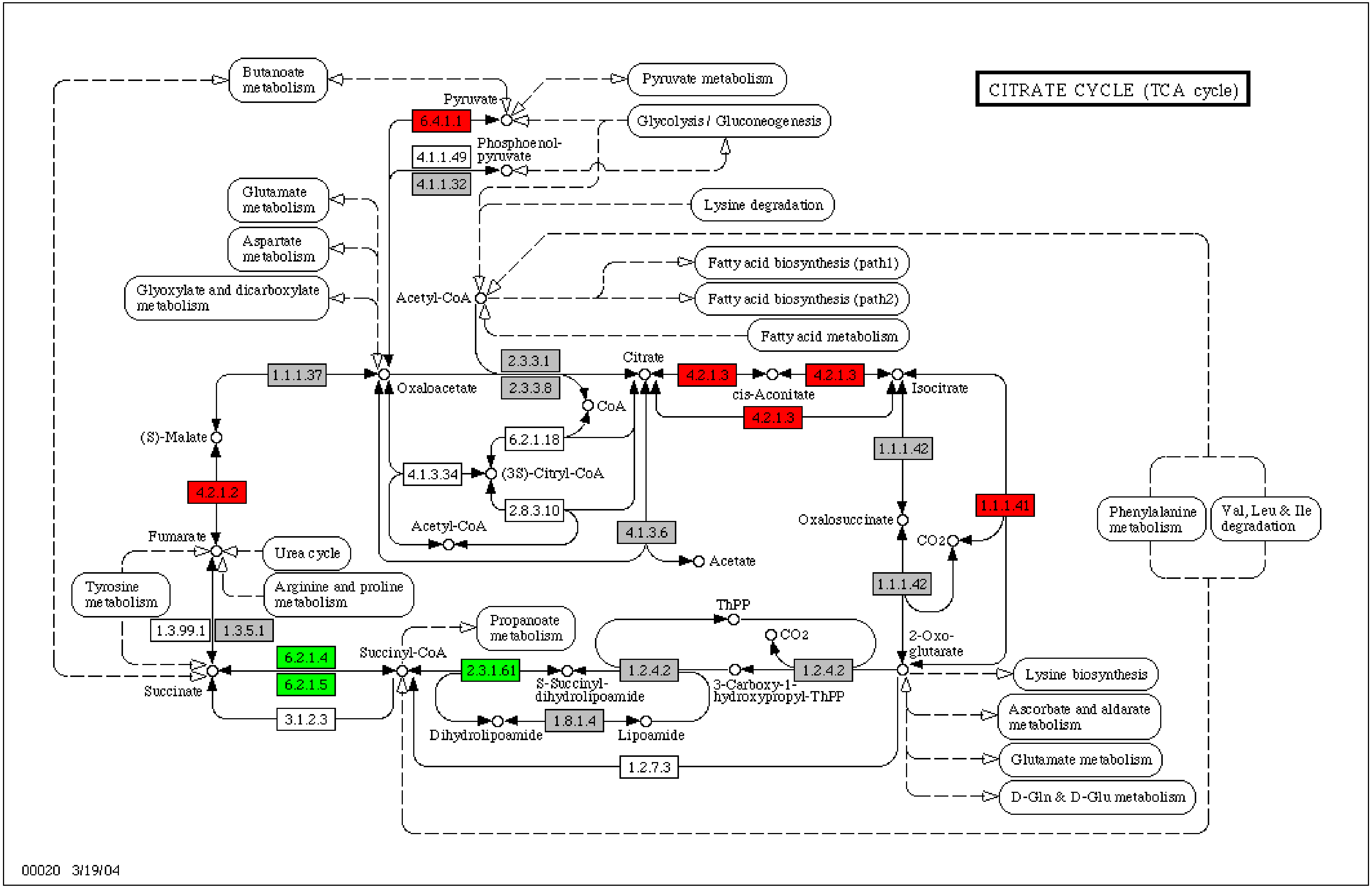

CITRATE CYCLE (TCA cycle)
Butanoate metabolism
Pyruvate metabolism
Pyruvate
6.4.1.1
Glycolysis / Gluconeogenesis
4.1.1.49
4.1.1.32
Phosphoenol-pyruvate
Glutamate metabolism
Aspartate metabolism
Glyoxylate and dicarboxylate metabolism
Lysine degradation
Fatty acid biosynthesis (path1)
Fatty acid biosynthesis (path2)
Fatty acid metabolism
Acetyl-CoA
1.1.1.37
Oxaloacetate
2.3.3.1
2.3.3.8
Citrate
4.2.1.3
cis-Aconitate
4.2.1.3
4.2.1.3
Isocitrate
CoA
6.2.1.18
(S)-Malate
4.1.3.34
(3S)-Citryl-CoA
1.1.1.42
4.2.1.2
2.8.3.10
1.1.1.41
Phenylalanine metabolism
Val, Leu & Ile degradation
Acetyl-CoA
4.1.3.6
Oxalosuccinate
CO2
Fumarate
Urea cycle
Acetate
Tyrosine metabolism
Arginine and proline metabolism
1.1.1.42
1.3.99.1
1.3.5.1
Propanoate metabolism
ThPP
CO2
6.2.1.4
6.2.1.5
Succinyl-CoA
2.3.1.61
1.2.4.2
1.2.4.2
2-Oxo-glutarate
S-Succinyl-dihydrolipoamide
3-Carboxy-1-hydroxypropyl-ThPP
Lysine biosynthesis
Succinate
3.1.2.3
1.8.1.4
Dihydrolipoamide
Lipoamide
Ascorbate and aldarate metabolism
Glutamate metabolism
1.2.7.3
D-Gln & D-Glu metabolism
00020 3/19/04

N1 group

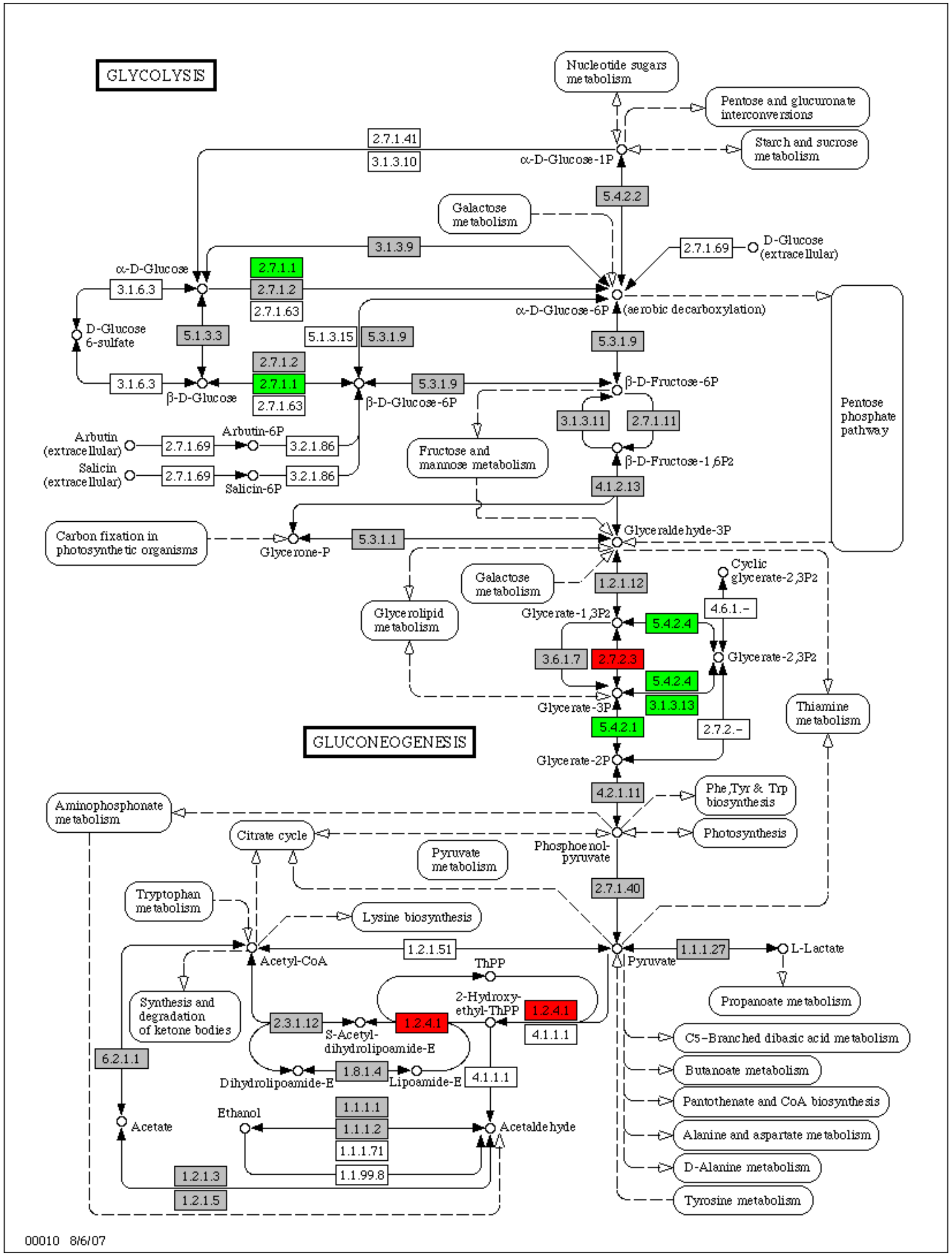
GLYCOLYSIS
GLUCONEOGENESIS
Nucleotide sugars metabolism
Pentose and glucuronate interconversions
Starch and sucrose metabolism
2.7.1.41
3.1.3.10
α-D-Glucose-1P
5.4.2.2
Galactose metabolism
3.1.3.9
2.7.1.69
D-Glucose (extracellular)
α-D-Glucose
2.7.1.1
2.7.1.2
2.7.1.63
3.1.6.3
D-Glucose 6-sulfate
5.1.3.3
5.1.3.15
5.3.1.9
α-D-Glucose-6P
(aerobic decarboxylation)
Pentose phosphate pathway
β-D-Glucose
β-D-Glucose-6P
β-D-Fructose-6P
3.1.3.11
2.7.1.11
β-D-Fructose-1,6P2
Arbutin (extracellular)
Arbutin-6P
3.2.1.86
Salicin (extracellular)
Salicin-6P
Fructose and mannose metabolism
4.1.2.13
Carbon fixation in photosynthetic organisms
5.3.1.1
Glycerone-P
Glyceraldehyde-3P
Galactose metabolism
1.2.1.12
Cyclic glycerate-2,3P2
4.6.1.-
Glycerolipid metabolism
Glycerate-1,3P2
5.4.2.4
3.6.1.7
2.7.2.3
Glycerate-2,3P2
Glycerate-3P
3.1.3.13
5.4.2.1
2.7.2.-
Thiamine metabolism
Glycerate-2P
4.2.1.11
Phe,Tyr & Trp biosynthesis
Aminophosphonate metabolism
Citrate cycle
Phosphoenol-pyruvate
Photosynthesis
Pyruvate metabolism
2.7.1.40
Tryptophan metabolism
Lysine biosynthesis
Acetyl-CoA
1.2.1.51
Pyruvate
1.1.1.27
L-Lactate
ThPP
2-Hydroxy-ethyl-ThPP
Synthesis and degradation of ketone bodies
2.3.1.12
1.2.4.1
4.1.1.1
Propanoate metabolism
S-Acetyl-dihydrolipoamide-E
C5-Branched dibasic acid metabolism
6.2.1.1
Dihydrolipoamide-E
1.8.1.4
Lipoamide-E
Butanoate metabolism
Ethanol
1.1.1.1
1.1.1.2
1.1.1.71
1.1.99.8
Acetaldehyde
Acetate
Pantothenate and CoA biosynthesis
Alanine and aspartate metabolism
1.2.1.3
1.2.1.5
D-Alanine metabolism
Tyrosine metabolism
00010 8/6/07

N1 group

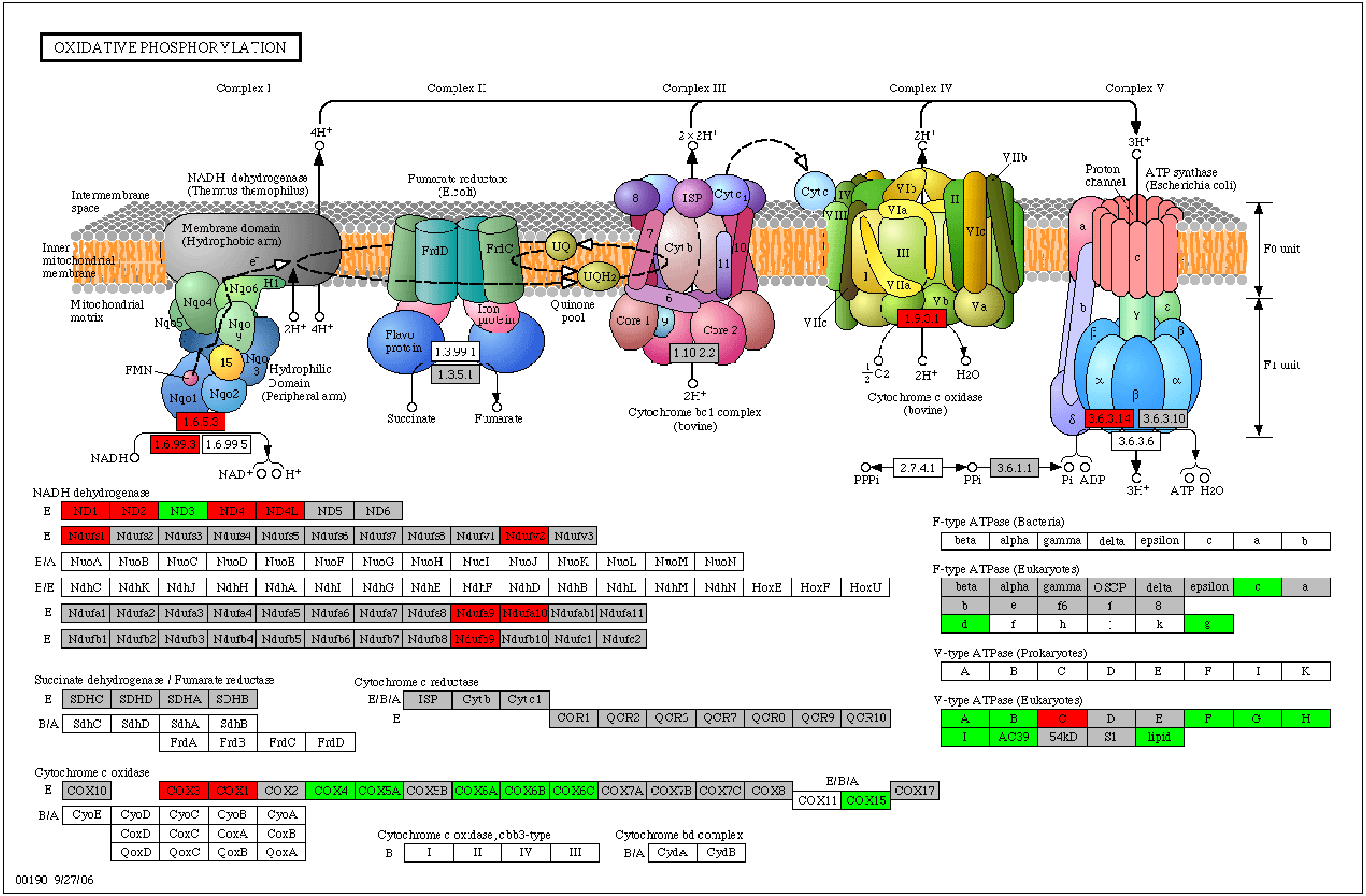
OXIDATIVE PHOSPHORYLATION
Complex I
Complex II
Complex III
Complex IV
Complex V
4H+
2×2H+
2H+
3H+
Intermembrane space
Inner mitochondrial membrane
Mitochondrial matrix
NADH dehydrogenase (Thermus themophilus)
Membrane domain (Hydrophobic arm)
Nqo4
Nqo6
H1
Nqo5
Nqo 9
15
Nqo 3
FMN
Nqo1
Nqo2
Hydrophilic Domain (Peripheral arm)
e-
2H+ 4H+
1.6.5.3
1.6.99.3
1.6.99.5
NADH
NAD+
H+
Fumarate reductase (E.coli)
FrdD
FrdC
Flavo protein
Iron protein
1.3.99.1
1.3.5.1
Succinate
Fumarate
UQ
UQH2
Quinone pool
8
ISP
Cyt c1
Cyt c
7
Cyt b
11
10
6
Core 1
9
Core 2
1.10.2.2
2H+
Cytochrome bc1 complex (bovine)
IV
VIII
VIb
VIa
VIIb
II
VIc
III
I
VIIa
Vb
Va
VIIc
1.9.3.1
1/2 O2
2H+
H2O
Cytochrome c oxidase (bovine)
Proton channel
ATP synthase (Escherichia coli)
a
c
b
γ
ε
β
α
δ
F0 unit
F1 unit
3.6.3.14
3.6.3.10
3.6.3.6
PPPi
2.7.4.1
PPi
3.6.1.1
Pi ADP
3H+
ATP H2O
NADH dehydrogenase
E ND1 ND2 ND3 ND4 ND4L ND5 ND6
E Ndufs1 Ndufs2 Ndufs3 Ndufs4 Ndufs5 Ndufs6 Ndufs7 Ndufs8 Ndufv1 Ndufv2 Ndufv3
B/A NuoA NuoB NuoC NuoD NuoE NuoF NuoG NuoH NuoI NuoJ NuoK NuoL NuoM NuoN
B/E NdhC NdhK NdhJ NdhH NdhA NdhI NdhG NdhE NdhF NdhD NdhB NdhL NdhM NdhN HoxE HoxF HoxU
E Ndufa1 Ndufa2 Ndufa3 Ndufa4 Ndufa5 Ndufa6 Ndufa7 Ndufa8 Ndufa9 Ndufa10 Ndufab1 Ndufa11
E Ndufb1 Ndufb2 Ndufb3 Ndufb4 Ndufb5 Ndufb6 Ndufb7 Ndufb8 Ndufb9 Ndufb10 Ndufc1 Ndufc2
Succinate dehydrogenase / Fumarate reductase
E SDHC SDHD SDHA SDHB
B/A SdhC SdhD SdhA SdhB
FrdA FrdB FrdC FrdD
Cytochrome c reductase
E/B/A ISP Cyt b Cyt c1
E COR1 QCR2 QCR6 QCR7 QCR8 QCR9 QCR10
Cytochrome c oxidase
E COX10 COX3 COX1 COX2 COX4 COX5A COX5B COX6A COX6B COX6C COX7A COX7B COX7C COX8
E/B/A COX11 COX15
COX17
B/A CyoE CyoD CyoC CyoB CyoA
CoxD CoxC CoxA CoxB
QoxD QoxC QoxB QoxA
Cytochrome c oxidase, cbb3-type
B I II IV III
Cytochrome bd complex
B/A CydA CydB
F-type ATPase (Bacteria)
beta alpha gamma delta epsilon c a b
F-type ATPase (Eukaryotes)
beta alpha gamma OSCP delta epsilon c a
b e f6 f 8
d f h j k g
V-type ATPase (Prokaryotes)
A B C D E F I K
V-type ATPase (Eukaryotes)
A B C D E F G H
I AC39 54kD S1 lipid
00190 9/27/06

N1 group

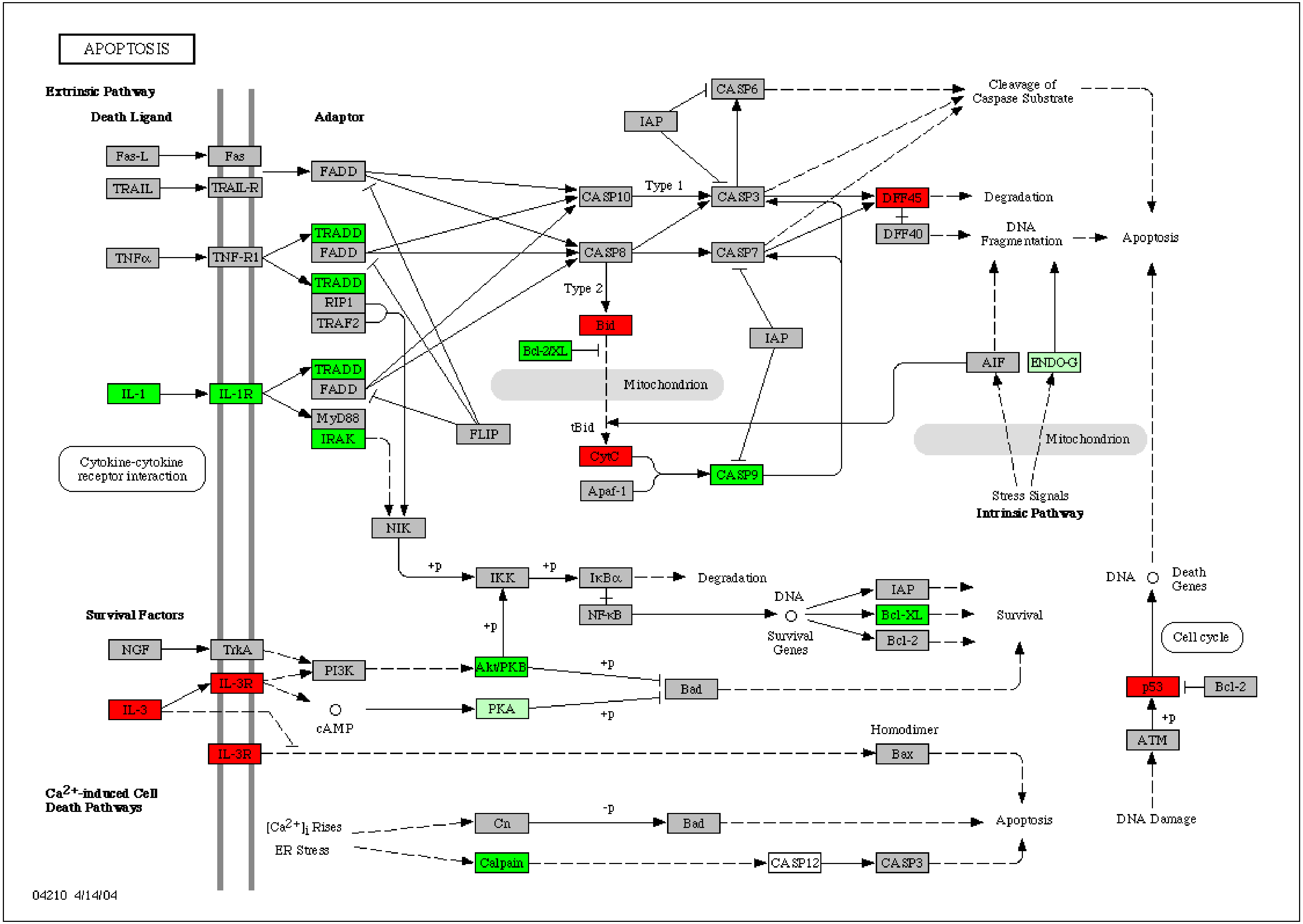
APOPTOSIS
Extrinsic Pathway
Death Ligand
Adaptor
Fas-L
Fas
TRAIL
TRAIL-R
FADD
TNFα
TNF-R1
TRADD
FADD
TRADD
RIP1
TRAF2
IL-1
IL-1R
TRADD
FADD
MyD88
IRAK
FLIP
Cytokine-cytokine receptor interaction
NIK
IKK
IκBα
NF-κB
+p
Degradation
CASP10
CASP8
Type 1
Type 2
CASP3
CASP6
CASP7
IAP
Cleavage of Caspase Substrate
DFF45
DFF40
Degradation
DNA Fragmentation
Apoptosis
Bid
Bcl-2/XL
Mitochondrion
tBid
CytC
Apaf-1
CASP9
AIF
ENDO-G
Stress Signals
Intrinsic Pathway
DNA
Survival Genes
Bcl-XL
Bcl-2
Survival
Death Genes
Cell cycle
Survival Factors
NGF
TrkA
PI3K
Akt/PKB
Bad
IL-3R
IL-3
cAMP
PKA
Homodimer
Bax
p53
ATM
DNA Damage
Ca2+-induced Cell Death Pathways
[Ca2+]i Rises
ER Stress
Cn
-p
Calpain
CASP12
04210 4/14/04

N1 group

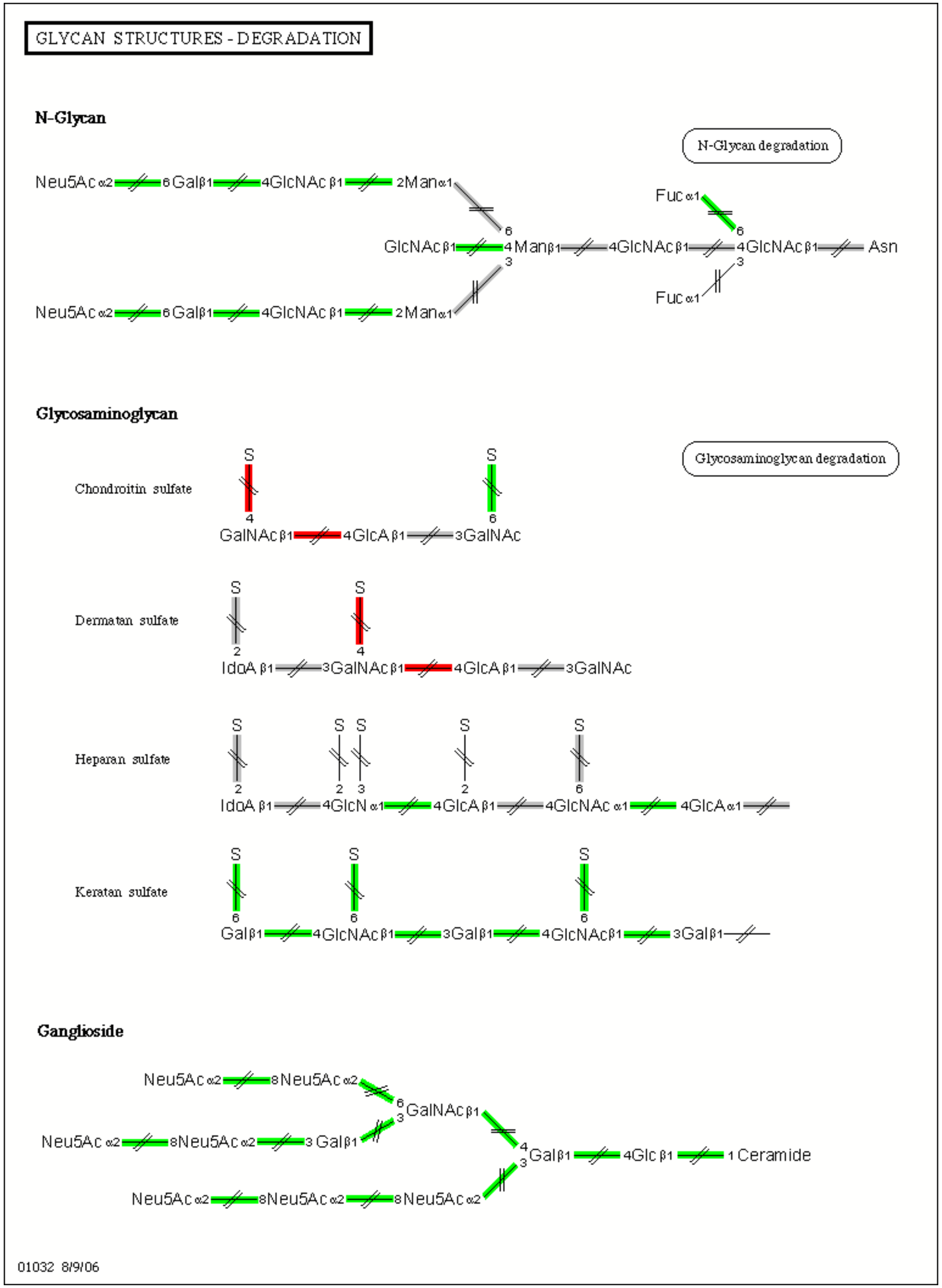
GLYCAN STRUCTURES - DEGRADATION
N-Glycan
N-Glycan degradation
Neu5Acα2 6Galβ1 4GlcNAcβ1 2Manα1
GlcNAcβ1 4Manβ1 4GlcNAcβ1 4GlcNAcβ1 Asn
Fucα1
Fucα1
Neu5Acα2 6Galβ1 4GlcNAcβ1 2Manα1
Glycosaminoglycan
Glycosaminoglycan degradation
Chondroitin sulfate
GalNAcβ1 4GlcAβ1 3GalNAc
Dermatan sulfate
IdoAβ1 3GalNAcβ1 4GlcAβ1 3GalNAc
Heparan sulfate
IdoAβ1 4GlcNα1 4GlcAβ1 4GlcNAcα1 4GlcAα1
Keratan sulfate
Galβ1 4GlcNAcβ1 3Galβ1 4GlcNAcβ1 3Galβ1
Ganglioside
Neu5Acα2 8Neu5Acα2
GalNAcβ1
Neu5Acα2 8Neu5Acα2 3 Galβ1
Galβ1 4Glcβ1 1Ceramide
Neu5Acα2 8Neu5Acα2 8Neu5Acα2
01032 8/9/06

N1 group

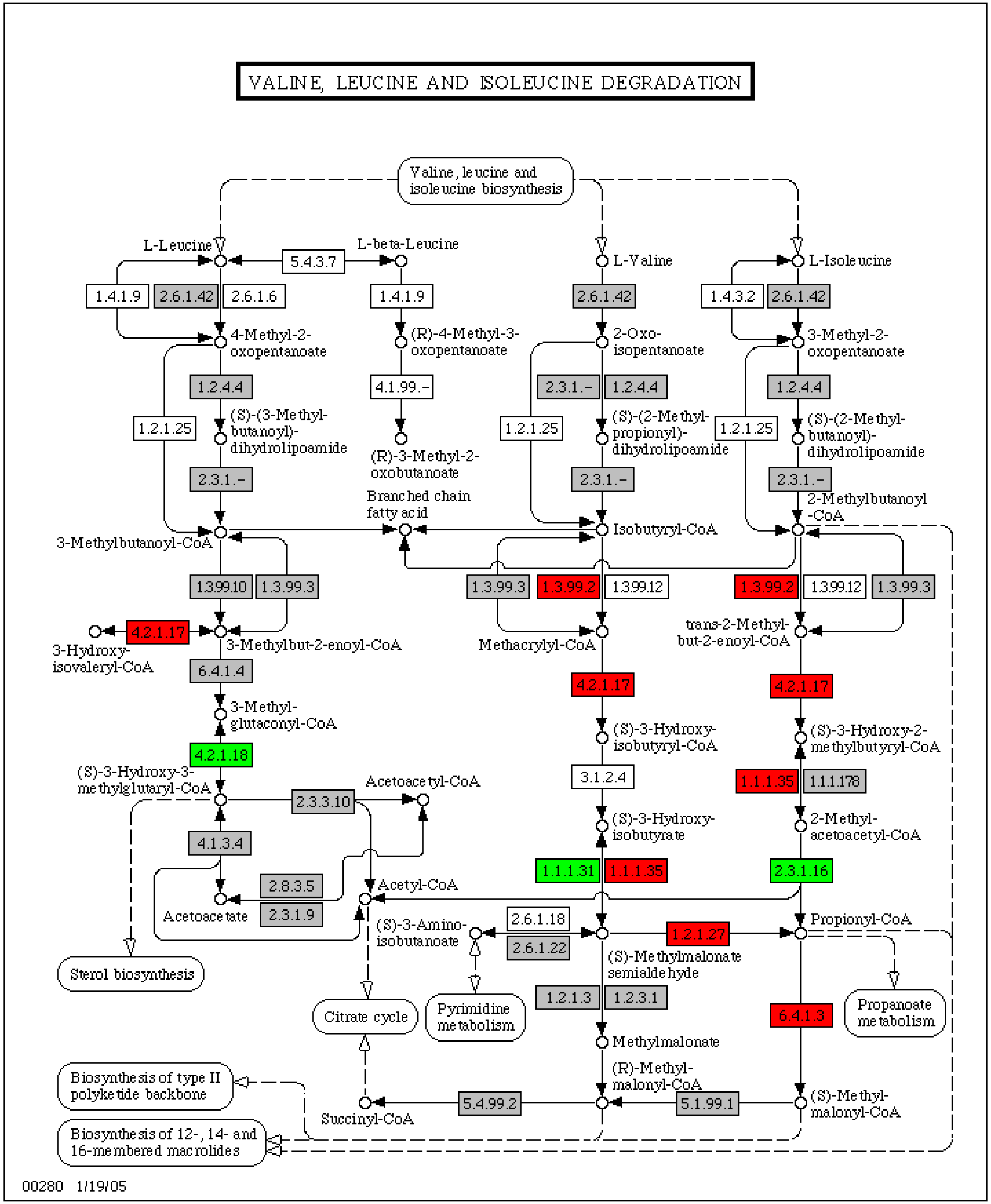

VALINE, LEUCINE AND ISOLEUCINE DEGRADATION
Valine, leucine and isoleucine biosynthesis
L-Leucine
L-beta-Leucine
L-Valine
L-Isoleucine
5.4.3.7
1.4.1.9
2.6.1.42
2.6.1.6
1.4.3.2
4-Methyl-2-oxopentanoate
(R)-4-Methyl-3-oxopentanoate
2-Oxo-isopentanoate
3-Methyl-2-oxopentanoate
1.2.4.4
4.1.99.-
2.3.1.-
1.2.1.25
(S)-(3-Methyl-butanoyl)-dihydrolipoamide
(R)-3-Methyl-2-oxobutanoate
(S)-(2-Methyl-propionyl)-dihydrolipoamide
(S)-(2-Methyl-butanoyl)-dihydrolipoamide
Branched chain fatty acid
2-Methylbutanoyl-CoA
3-Methylbutanoyl-CoA
Isobutyryl-CoA
1.3.99.10
1.3.99.3
1.3.99.2
1.3.99.12
3-Hydroxy-isovaleryl-CoA
4.2.1.17
3-Methylbut-2-enoyl-CoA
Methacrylyl-CoA
trans-2-Methyl-but-2-enoyl-CoA
6.4.1.4
3-Methyl-glutaconyl-CoA
(S)-3-Hydroxy-isobutyryl-CoA
(S)-3-Hydroxy-2-methylbutyryl-CoA
4.2.1.18
(S)-3-Hydroxy-3-methylglutaryl-CoA
2.3.3.10
Acetoacetyl-CoA
3.1.2.4
1.1.1.35
1.1.1.178
(S)-3-Hydroxy-isobutyrate
2-Methyl-acetoacetyl-CoA
4.1.3.4
2.8.3.5
2.3.1.9
Acetoacetate
Acetyl-CoA
1.1.1.31
2.3.1.16
(S)-3-Amino-isobutanoate
2.6.1.18
2.6.1.22
1.2.1.27
Propionyl-CoA
(S)-Methylmalonate semialdehyde
Sterol biosynthesis
Citrate cycle
Pyrimidine metabolism
1.2.1.3
1.2.3.1
Methylmalonate
6.4.1.3
Propanoate metabolism
(R)-Methyl-malonyl-CoA
Biosynthesis of type II polyketide backbone
5.4.99.2
5.1.99.1
(S)-Methyl-malonyl-CoA
Succinyl-CoA
Biosynthesis of 12-, 14- and 16-membered macrolides
00280 1/19/05

N1 group

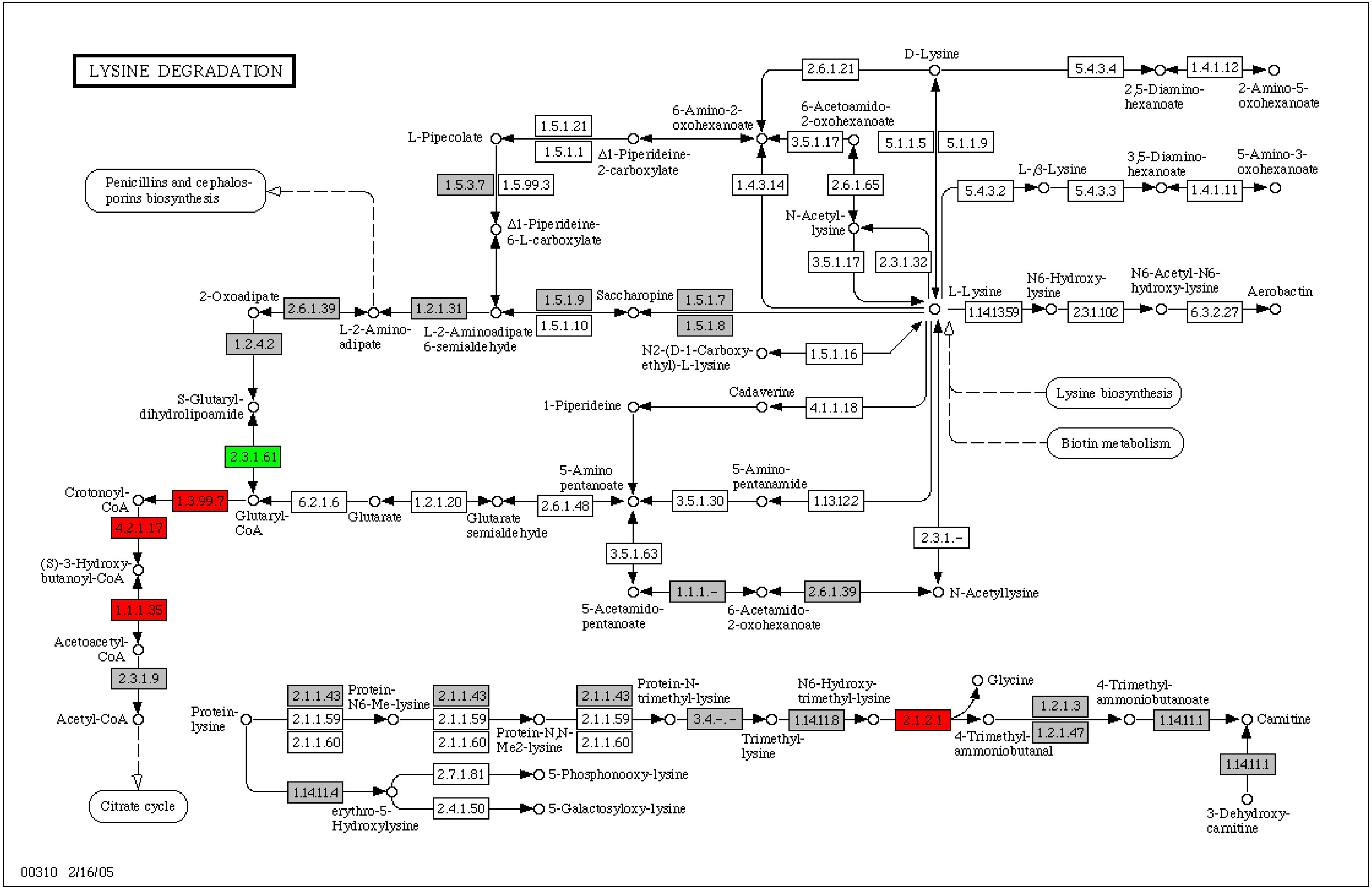
LYSINE DEGRADATION
Penicillins and cephalosporins biosynthesis
L-Pipecolate
1.5.1.21
1.5.1.1
Δ1-Piperideine-2-carboxylate
6-Amino-2-oxohexanoate
2.6.1.21
D-Lysine
5.4.3.4
2,5-Diaminohexanoate
1.4.1.12
2-Amino-5-oxohexanoate
6-Acetoamido-2-oxohexanoate
3.5.1.17
5.1.1.5
5.1.1.9
1.5.3.7
1.5.99.3
1.4.3.14
2.6.1.65
L-β-Lysine
5.4.3.2
5.4.3.3
3,5-Diaminohexanoate
1.4.1.11
5-Amino-3-oxohexanoate
Δ1-Piperideine-6-L-carboxylate
N-Acetyl-lysine
3.5.1.17
2.3.1.32
2-Oxoadipate
2.6.1.39
L-2-Aminoadipate
1.2.1.31
L-2-Aminoadipate 6-semialdehyde
1.5.1.9
1.5.1.10
Saccharopine
1.5.1.7
1.5.1.8
L-Lysine
1.14.13.59
N6-Hydroxy-lysine
2.3.1.102
N6-Acetyl-N6-hydroxy-lysine
6.3.2.27
Aerobactin
1.2.4.2
N2-(D-1-Carboxyethyl)-L-lysine
1.5.1.16
S-Glutaryl-dihydrolipoamide
1-Piperideine
Cadaverine
4.1.1.18
Lysine biosynthesis
Biotin metabolism
2.3.1.61
5-Amino pentanoate
5-Amino-pentanamide
Crotonoyl-CoA
1.3.99.7
Glutaryl-CoA
6.2.1.6
Glutarate
1.2.1.20
Glutarate semialdehyde
2.6.1.48
3.5.1.30
1.13.12.2
4.2.1.17
2.3.1.-
(S)-3-Hydroxy-butanoyl-CoA
3.5.1.63
1.1.1.35
1.1.1.-
2.6.1.39
N-Acetyllysine
5-Acetamido-pentanoate
6-Acetamido-2-oxohexanoate
Acetoacetyl-CoA
2.3.1.9
Acetyl-CoA
Protein-lysine
2.1.1.43
2.1.1.59
2.1.1.60
Protein-N6-Me-lysine
Protein-N,N-Me2-lysine
Protein-N-trimethyl-lysine
3.4.-.-
Trimethyl-lysine
N6-Hydroxy-trimethyl-lysine
1.14.11.8
2.1.2.1
Glycine
4-Trimethyl-ammoniobutanal
1.2.1.3
1.2.1.47
4-Trimethyl-ammoniobutanoate
1.14.11.1
Carnitine
3-Dehydroxy-carnitine
Citrate cycle
1.14.11.4
erythro-5-Hydroxylysine
2.7.1.81
5-Phosphonooxy-lysine
2.4.1.50
5-Galactosyloxy-lysine
00310 2/16/05

N1 group

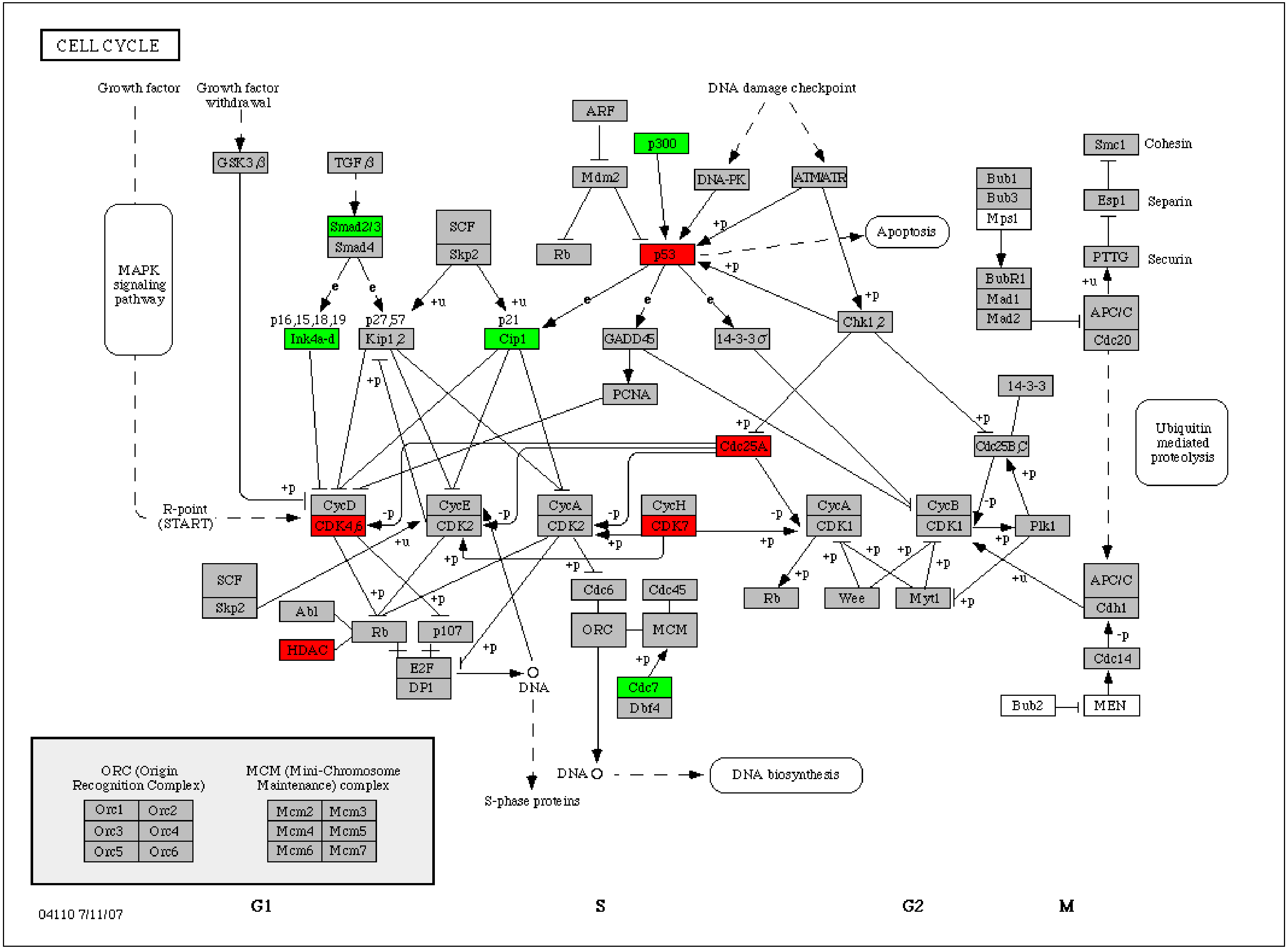
CELL CYCLE
Growth factor
Growth factor withdrawal
GSK3β
TGFβ
Smad2/3
Smad4
SCF
Skp2
ARF
Mdm2
p300
DNA damage checkpoint
DNA-PK
ATM/ATR
Rb
p53
Apoptosis
MAPK signaling pathway
p16,15,18,19
Ink4a-d
p27,57
Kip1,2
p21
Cip1
GADD45
14-3-3σ
Chk1,2
PCNA
Cdc25A
14-3-3
Cdc25B,C
Bub1
Bub3
Mps1
BubR1
Mad1
Mad2
Smc1
Cohesin
Esp1
Separin
PTTG
Securin
APC/C
Cdc20
Ubiquitin mediated proteolysis
R-point (START)
CycD
CDK4,6
CycE
CDK2
CycA
CDK2
CycH
CDK7
CycA
CDK1
CycB
CDK1
Plk1
SCF
Skp2
Abl
HDAC
Rb
p107
E2F
DP1
DNA
Cdc6
Cdc45
ORC
MCM
Cdc7
Dbf4
Rb
Wee
Myt1
APC/C
Cdh1
Cdc14
Bub2
MEN
DNA
S-phase proteins
DNA biosynthesis
ORC (Origin Recognition Complex)
Orc1
Orc2
Orc3
Orc4
Orc5
Orc6
MCM (Mini-Chromosome Maintenance) complex
Mcm2
Mcm3
Mcm4
Mcm5
Mcm6
Mcm7
04110 7/11/07
G1
S
G2
M

N1 group

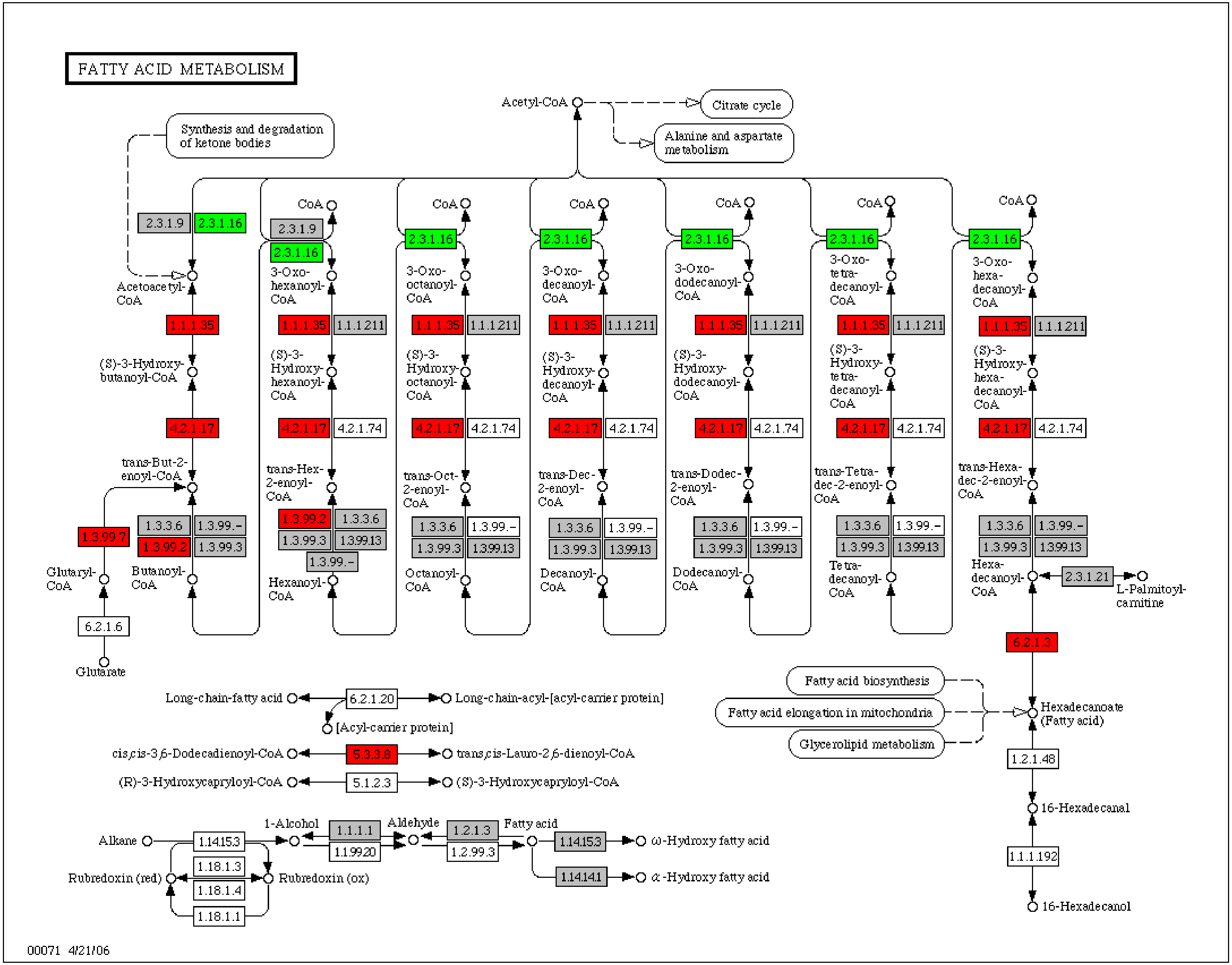
FATTY ACID METABOLISM
Acetyl-CoA
Citrate cycle
Alanine and aspartate metabolism
Synthesis and degradation of ketone bodies
CoA
2.3.1.9
2.3.1.16
Acetoacetyl-CoA
3-Oxo-hexanoyl-CoA
3-Oxo-octanoyl-CoA
3-Oxo-decanoyl-CoA
3-Oxo-dodecanoyl-CoA
3-Oxo-tetra-decanoyl-CoA
3-Oxo-hexa-decanoyl-CoA
1.1.1.35
1.1.1.211
(S)-3-Hydroxy-butanoyl-CoA
(S)-3-Hydroxy-hexanoyl-CoA
(S)-3-Hydroxy-octanoyl-CoA
(S)-3-Hydroxy-decanoyl-CoA
(S)-3-Hydroxy-dodecanoyl-CoA
(S)-3-Hydroxy-tetra-decanoyl-CoA
(S)-3-Hydroxy-hexa-decanoyl-CoA
4.2.1.17
4.2.1.74
trans-But-2-enoyl-CoA
trans-Hex-2-enoyl-CoA
trans-Oct-2-enoyl-CoA
trans-Dec-2-enoyl-CoA
trans-Dodec-2-enoyl-CoA
trans-Tetra-dec-2-enoyl-CoA
trans-Hexa-dec-2-enoyl-CoA
1.3.99.7
1.3.3.6
1.3.99.-
1.3.99.2
1.3.99.3
1.3.99.13
Glutaryl-CoA
Butanoyl-CoA
Hexanoyl-CoA
Octanoyl-CoA
Decanoyl-CoA
Dodecanoyl-CoA
Tetra-decanoyl-CoA
Hexa-decanoyl-CoA
2.3.1.21
L-Palmitoyl-carnitine
6.2.1.6
Glutarate
6.2.1.3
Long-chain-fatty acid
6.2.1.20
Long-chain-acyl-[acyl-carrier protein]
[Acyl-carrier protein]
cis,cis-3,6-Dodecadienoyl-CoA
5.3.3.8
trans,cis-Lauro-2,6-dienoyl-CoA
(R)-3-Hydroxycapryloyl-CoA
5.1.2.3
(S)-3-Hydroxycapryloyl-CoA
Fatty acid biosynthesis
Fatty acid elongation in mitochondria
Glycerolipid metabolism
Hexadecanoate (Fatty acid)
1.2.1.48
16-Hexadecanal
1.1.1.192
16-Hexadecanol
Alkane
1.14.15.3
1.18.1.3
1.18.1.4
1.18.1.1
Rubredoxin (red)
Rubredoxin (ox)
1-Alcohol
1.1.1.1
1.1.99.20
Aldehyde
1.2.1.3
1.2.99.3
Fatty acid
1.14.15.3
ω-Hydroxy fatty acid
1.14.14.1
α-Hydroxy fatty acid
00071 4/21/06
